# Integrative In Silico and In Vitro Screening of Low Molecular Weight Compounds Targeting SARS‐CoV‐2 RNA Elements

**DOI:** 10.1002/cbic.202500668

**Published:** 2025-11-29

**Authors:** Sabrina Toews, Betül Ceylan, Anna Wacker, Megan Ken, Harald Schwalbe

**Affiliations:** ^1^ Institute for Organic Chemistry and Chemical Biology Goethe University Frankfurt Max‐von‐Laue‐Strasse 7 60438 Frankfurt am Main Germany; ^2^ Center of Biomolecular Magnetic Resonance (BMRZ) Goethe University Frankfurt Max‐von‐Laue‐Strasse 9 60438 Frankfurt am Main Germany; ^3^ Department of Integrative Structural and Computational Biology The Scripps Research Institute 10660 North Torrey Pines Road La Jolla CA 92037 USA

**Keywords:** drug discovery, nuclear magnetic resonance spectroscopy, RNA, SARS‐CoV‐2, virtual screening

## Abstract

An approach combining virtual and nuclear magnetic resonance (NMR)‐based screening is presented here to identify low molecular weight molecules (small molecules) targeting viral RNA elements from the SARS‐CoV‐2 genome. The so‐called high‐resolution RNA structural Fragment Assembly of RNA with Full‐Atom Refinement 2 (FARFAR2) ensembles of the conserved 5^′^‐terminal stem‐loop 1 (SL1) and the pseudoknot of the –1 programed frameshift element are used as targets for binding of small molecules of three different virtual libraries of compounds. The resulting hits predicted by virtual screening are probed for their binding to the two RNA elements by ligand‐ and target‐based NMR experiments. The results demonstrate that the integration of virtual and experimental NMR screening efficiently identifies RNA‐binding small molecules as start molecular entities to advance RNA‐targeted antiviral therapies in an efficient manner. The strategy does not only apply to SARS‐CoV‐2, but also provides a rapid, highly specific route to discovering therapeutics for other RNA‐based pathogens, highlighting the critical role of RNA structural data in enriching virtual drug discovery efforts.

## Graphical abstract

A combined virtual and nuclear magnetic resonance (NMR)‐based screening approach identifies small molecules that bind conserved SARS‐CoV‐2 RNA structures. Diverse compound libraries are screened using full‐atom refinement 2‐generated ensembles of stem‐loop 1 and the frameshift pseudoknot. Predicted hits are validated by ligand‐ and target‐detected NMR, and preliminary structure‐activity insights highlight determinants of binding. This strategy provides an efficient, broadly applicable method for discovering RNA‐targeted antiviral candidates.

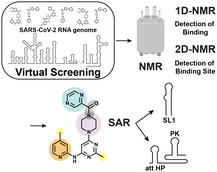

## 1 Introduction

The global health crisis caused by the Severe Acute Respiratory Syndrome Coronavirus 2 (SARS‐CoV‐2) has underscored the crucial need for the development of rapid antiviral medication. While most drugs, low molecular weightinhibitors, as a therapeutic approach have focused on viral proteins, the highly structured RNA genome of SARS‐CoV‐2 represents an underexplored but promising target space for these low molecular weight compounds (small molecule) intervention. At first glance, this underrepresentation is surprising as structured RNA elements are highly conserved and play essential roles throughout viral life cycles, including genome replication, transcription, and translation. Importantly, SARS‐CoV‐2 provides the opportunity for a holistic antiviral approach. With only 29 viral proteins and, as defined in our previous work, 15 conserved structured RNA elements, it has a dual, comparatively small target space.^[^ ^1^ ^,^ ^2^ ^]^

Recent advances in RNA structural biology have enabled structure‐based discovery of RNA‐targeted small molecules. Methods such as Selective 2^′^‐Hydroxyl Acylation analyzed by Primer Extension (SHAPE) probing and platforms like Inforna 2.0 facilitate mapping and targeting of functional RNA elements, including viral RNAs such as the HIV‐1 trans‐activation response (TAR) element and SARS‐CoV‐2 motifs.^[^ ^3^, ^4^, ^5^, ^6^, ^7^ ^]^ In parallel, studies on viral noncoding RNAs have revealed that structural and chemical features render these elements highly amenable to small molecule targeting, as shown in investigations of HIV, hepatitis C virus (HCV), and coronaviruses.^[^ ^8^ ^]^ Recent work highlights the therapeutic potential of structured RNAs as drug targets and provide a compelling framework for developing antivirals that disrupt essential RNA‐mediated processes in SARS‐CoV‐2.^[^ ^9^ ^]^

**Figure 1:**
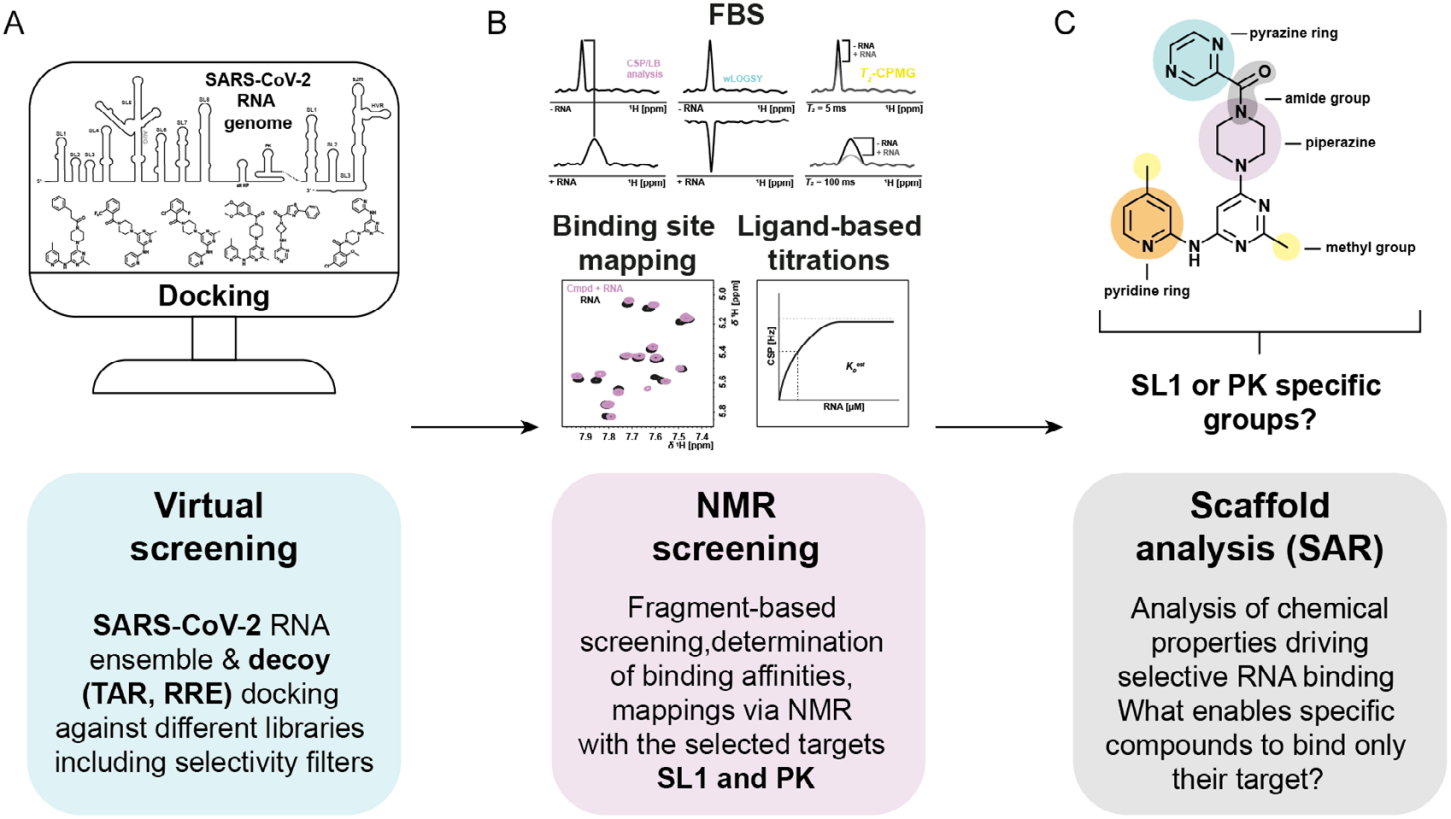
Workflow for targeting SARS‐CoV‐2‐derived RNA elements with compounds of low molecular weight. A) Virtual screening of the SARS‐CoV‐2 RNA genome ensemble and the HIV decoy RNAs (TAR, RRE) against three known compound libraries (68.067 compounds in total) including diverse filters to enhance the computed selectivity.^[^ ^10^ ^]^ B) Severe filtering of the compounds led to a significant reduction in the number of prioritized hits and target prioritization of the RNA elements SL1 and PK, enabling a follow‐up study via NMR to detect and quantify RNA‐ligand interactions. NMR experiments included ligand‐ and RNA‐detected measurements, which allowed any interaction to be determined, binding affinities to be estimated, and the interaction sites to be identified. C) A structure–activity relationship (SAR) analysis was performed as the final step to identify functional groups of binders that drive the interaction with either SL1 or PK.

To leverage this potential, we conducted an in silico virtual screening (VS) campaign across the published SARS‐CoV‐2 RNA Fragment Assembly of RNA with Full‐Atom Refinement 2 (FARFAR2) ensemble.^[^ ^10^ ^]^ This genome‐wide approach allowed the identification and filtering of druggable RNAs and their capability to bind small molecules prior to experimental validation of proposed binders (**Figure** **1**A). Unlike static models, RNA ensembles such as those generated by FARFAR2 represent a diverse set of energetically plausible conformations, enabling identification of ligands that may bind to alternative or less‐populated states.^[^ ^11^ ^]^ This approach builds on recent successes in large‐scale docking screens and fragment‐based discovery.^[^ ^12^ ^,^ ^13^ ^]^ Ultralarge structure‐based docking screens of 235 million compounds identified noncovalent main protease (M^pro^) inhibitors with nanomolar affinity, which were validated by crystallography and demonstrated broad‐spectrum antiviral activity.^[^ ^14^ ^]^ To validate hits predicted by VS against dynamic RNA ensembles, we employ nuclear magnetic resonance (NMR) spectroscopy, a core method in early‐stage drug discovery particularly for challenging targets such as RNA for which fragment‐RNA complexes cannot be crystallized (Figure 1B). Fragment‐based screening (FBS) using NMR enables the detection of weak and transient interactions that are otherwise undetectable.^[^ ^15^ ^,^ ^16^ ^]^ Complementary fragment‐based NMR binding sites mapping across 25 SARS‐CoV‐2 proteins, revealed conserved chemotypes for structure‐guided optimization.^[^ ^17^ ^]^ We here combined virtual and NMR screening to yield a set of chemically diverse fragments that either bound selectively to the 5^′^‐untranslated region (UTR) stem‐loop 1 (SL1) and the frameshift‐stimulatory pseudoknot (PK), or nonselectively to both RNAs. Even without chemical optimization, comparative analysis of the fragment hits allowed us to extract preliminary structure–activity relationships (SAR) (Figure 1C).

**Figure 2:**
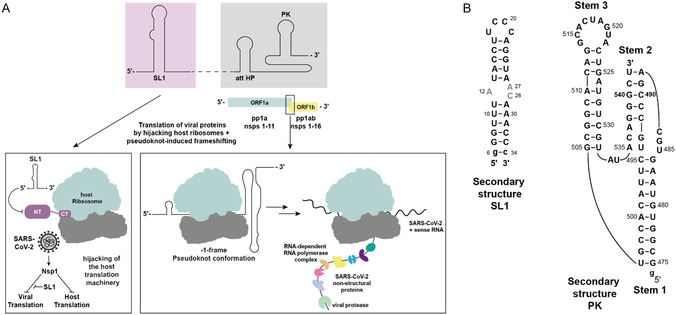
Mechanism of translation hijacking and ribosomal frameshifting in SARS‐CoV‐2. A) Genomic context of the SARS‐CoV‐2 RNA elements SL1 and PK. SL1 is located in the 5^′^‐UTR (lilac), while the frameshifting element (FSE), which includes the PK, is located between the open reading frames ORF1a and ORF1b (gray). The proposed mechanisms of action for SL1 (left) and PK (right) are illustrated. B) The secondary structure of SL1 comprises an internal loop formed by the residues A12, A27, and A28 and a UCCC apical loop. PK adopts a compact H‐type PK architecture composed of three stems (S1‐S3).

Among potential SARS‐CoV‐2 RNA targets, SL1 at the 5^′^‐terminal end and a central PK, part of the programed –1 frameshift element of the coding part of the RNA genome, have emerged as key druggable RNA elements (**Figure** **2**A). SL1 interacts with the nonstructural protein 1 (Nsp1) to promote viral translation by displacing Nsp1 from the host ribosome, thus, making it a promising therapeutic target.^[^ ^18^, ^19^, ^20^, ^21^, ^22^ ^]^ SL1 adopts a conserved secondary structure comprising two A‐form helices flanking a pyrimidine‐rich apical loop and an asymmetric internal loop with a 1 × 2 nucleotide configuration (Figure 2B). This internal loop serves as binding site for small molecules and is stabilized by a specific transient interaction between the adenosine 12 (A12) and the cytidine 28 (C28), which form an A^+^•C wobble base pair.^[^ ^23^ ^]^ The PK induces programed –1 ribosomal frameshifting, a critical mechanism including expression of the viral replicase.^[^ ^24^ ^,^ ^25^ ^]^ The SARS‐CoV‐2 PK adopts a distinctive three‐stem structure (S1‐S3) connected by loops L1‐L3 (Figure 2B).

S1 forms just after the slippery sequence and stacks with S2, which arises from base pairing between the S1 loop and a downstream region; S3 originates from the S1‐S2 loop and folds into S1's major groove, enhancing PK stability. L2 is particularly critical for viral growth rates, while L3 differs between SARS‐CoV‐1 and SARS‐CoV‐2 in a single C533A mutation.^[^ ^26^, ^27^, ^28^ ^]^ Condensed, this work explores SL1 and the PK as representative SARS‐CoV‐2 RNA targets. We evaluate the compound‐binding capabilities of SL1 and the PK, building on previous work by Sreeramulu, Richter et al.^[^ ^29^ ^]^ However, as with many RNA‐targeted ligands, selectivity remains a central concern given the structural similarities among different RNA motifs and the risk of off‐target binding. We here investigated this selectivity by examining two completely different SARS‐CoV‐2 RNA elements in parallel.

## 2 Results and Discussion

### 2.1 Virtual Screening of the SARS‐CoV‐2 Genome Identified SL1 and PK as Key Targetable RNA Elements

**Figure 3:**
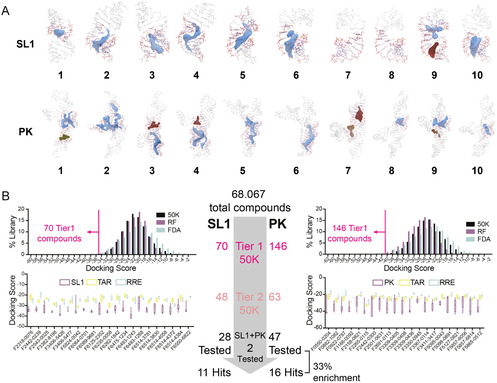
Structure‐based virtual screening against SARS‐CoV‐2 RNA ensembles. A) Structures of the SL1 and PK ensemble conformers and the pockets used for receptor generation. For each ensemble, the top 10 most energetically favorable conformers were chosen and further processed via the ICM Pocketfinder function to determine the most favorable location for small molecule binding.^[^ ^30^ ^]^ Pockets were defined to be residues within 5 Å of the colored region(s), and these selected residues are shown in color with the rest of the RNA in gray. B) The docked compounds were filtered based on docking score thresholds and then redocked against the TAR and RRE decoy ensembles of HIV for selectivity. Those passing the decoy filters were then tested for selectivity against other viral RNA targets, yielding 134 candidates for in vitro testing. 77 of the 134 compounds passed the subsequent quality control (QC). The validated sublibrary includes 28 SL1 specific binders (S), 47 PK specific binders (PK), and 2 compounds (PS) that showed in silico binding to both RNA targets.

To identify potential small molecules targeting SARS‐CoV‐2 RNA elements, we conducted a structure‐based VS campaign against seven RNA ensembles of the viral genome (5^′^‐UTR: SL1, SL2, SL4, SL5; PK; 3^′^‐UTR: SL1 and SL2), revealing molecules with favorable binding affinities and interaction profiles.^[^ ^10^ ^]^ We docked our libraries against ten conformers of each ensemble, with docking pockets determined using ICM PocketFinder, a built‐in tool that calculates the most favorable binding sites and has been validated in previous studies for detecting druggable regions in RNA (**Figure** **3**A, Figure 1, Supporting Information).^[^ ^13^ ^,^ ^30^, ^31^, ^32^ ^]^ Three compound libraries were expanded by calculating protonation sites at pH = 5.4, 7.4, and 9.4, resulting in a 50.000‐compound (50K) diversity library (60.985 total), an RNA‐focused library (4871 total) from Life Chemicals, Inc., and an FDA‐approved drug library (2211 total) downloaded from the ZINC database.^[^ ^33^ ^]^ In Tier0, the 50K library was docked twice per ensemble, while the FDA and RNA‐focused libraries were docked five times (or twice for selected ensembles), enabling a comprehensive assessment of ligand binding across diverse RNA target conformations.

To enrich selective binders for testing in vitro, we subjected the VS results to rigorous filtering. For each of the three libraries, we removed any positive value scores, which refer to unfavorable or unlikely binding events, and then chose the top score for each compound from the docking replicates. Compounds were retained only if their docking scores were at least three standard deviations below the mean of the respective library (Figure 2, Supporting Information). After filtering, SL1, PK and SL5 ensembles contained the highest number of remaining hits with favorable scores, which were selected for subsequent experimental validation. The heavily reduced libraries were subsequently redocked ten times against their original target and both HIV decoy ensembles TAR and rev‐response element (RRE) (Tier1). Surprisingly, only six compounds passed the selectivity filter for SL5, compared to 72 for PK and 53 for SL1. This finding suggests that some genomic elements may have a higher capacity for selective binding than others. We moved forward with one more selectivity test with SL1 and PK. While TAR and RRE are appropriate decoys representing general A‐form helices with simple elements found in the transcriptome, we further evaluated the selectivity among the SARS‐CoV‐2 genomic elements themselves. With the remaining 50K compounds that passed the selectivity filter for the PK (63) and SL1 (48) ensembles, we redocked against the other SARS‐CoV‐2 RNA ensembles. PK had 30 compounds passed this selectivity filter and SL1 had eight (Tier3). We then purchased a subset of 134 molecules from Tier2 and Tier3 for PK and SL1 to test for binding in vitro (Figure 3B).

### 2.2 NMR Validation of RNA‐Ligand Interactions through FBS Enabled Selective Refinement of Candidate Compounds

**Figure 4:**
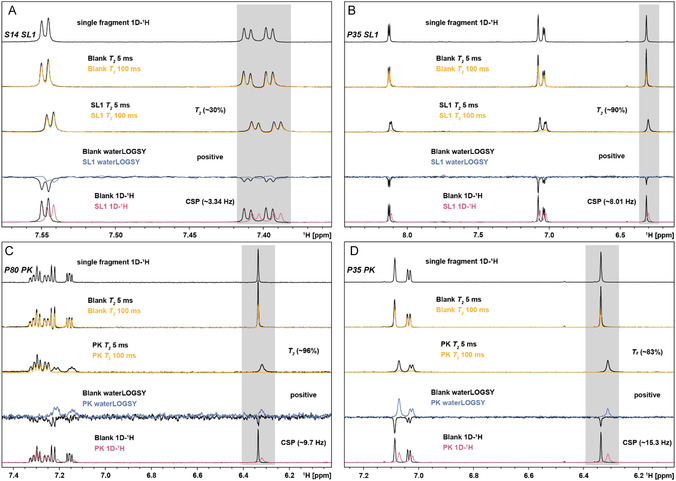
NMR‐based fragment screening of the target RNAs SL1 and PK. Overlay of 1D‐^1^H NMR, waterLOGSY, and *T*_*2*_ relaxation spectra (relaxation delays: 5 ms and 100 ms). “Blank” spectra refer to those recorded in the absence of target RNA. Signals used for interaction evaluation are highlighted with gray boxes. NMR screening of the compounds A) S14 and B) P35 in the presence of SL1, and C) P80 and D) P35 in the presence of PK.

**Figure 5:**
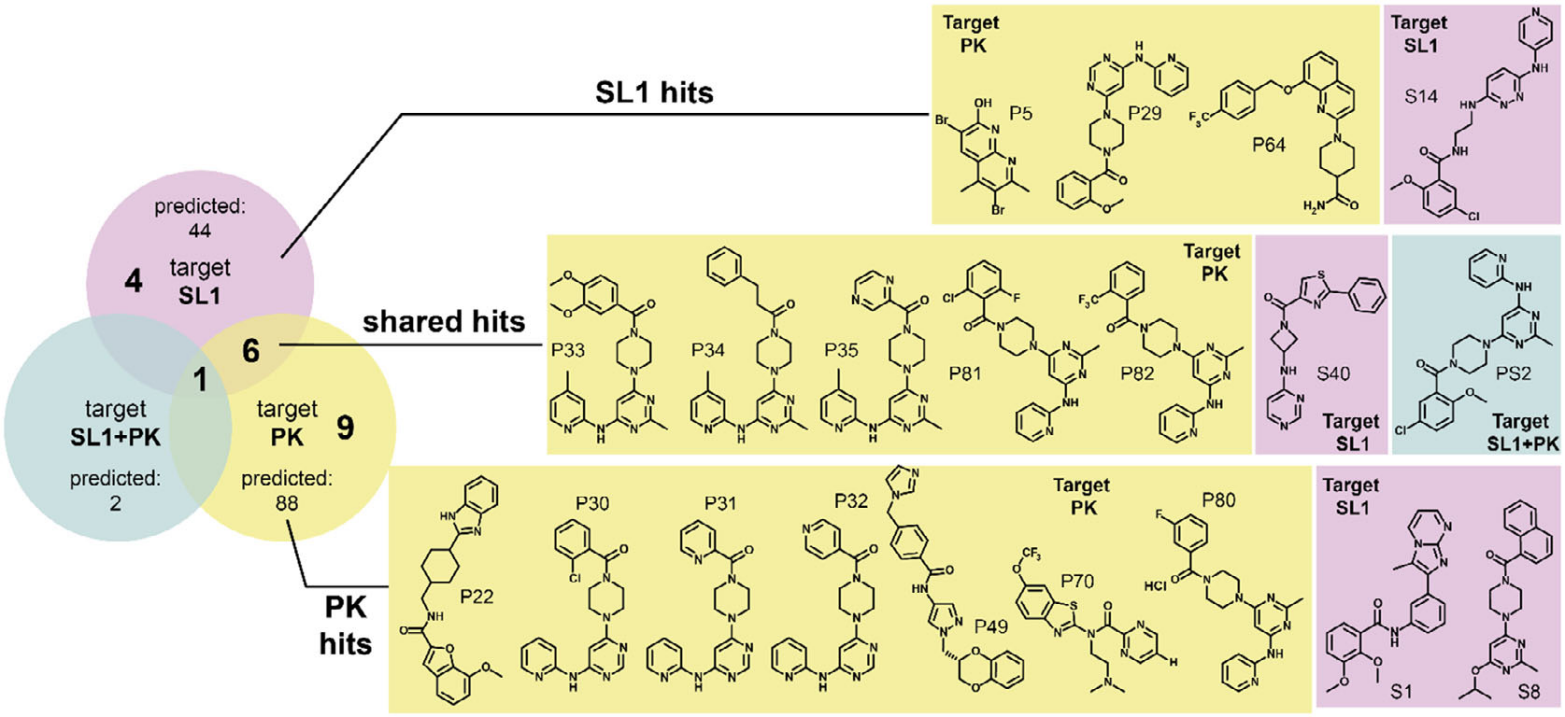
Summary of compounds hits identified for the RNA targets SL1 and PK. The Venn diagram (left) illustrates the overlap of hit compounds identified by NMR. Four, nine, and seven compounds were found to bind exclusively to SL1, PK, or both, respectively. Structures of hit compounds are color‐coded according to their virtual screening predicted target: SL1 (lilac), PK (yellow), and SL1 + PK (blue).

To validate the VS‐derived small molecule‐RNA binding for the two selected RNA elements SL1 and PK, NMR screening was conducted with purchased compounds and after they passed an NMR‐based quality control (QC), including checking for purity and solubility in the used NMR buffer (77 out of 134) (Table 1, Supporting Information). In general, the experimental setup included: 1) single compound screening, 2) NMR‐based determination of binding affinities, and 3) RNA‐detected NMR experiments for the binding mode characterization. To simultaneously assess binding and target selectivity, the 77 compounds were measured in the presence of either SL1 or PK, irrespective of the predicted target, in a compound‐to‐target ratio of 20:1 (**Figure** **4**, Table 1, Supporting Information). The results were evaluated as previously reported by Sreeramulu, Richter et al.^[^ ^29^ ^]^ For clarity, we have numbered the compounds consecutively and labeled them with the indices “P” for those computed to bind PK, “S” for those computed to bind SL1 in the VS, and “PS” for those computed to interact with both targets. In general, we considered compounds to be hits if two out of three of the applied experiments resulted in a clear effect: 1) line‐broadening (LB), 2) chemical shift perturbations (CSPs) above 3 Hz, 3) waterLOGSY factor of ≈1. NMR screening results were ranked for each target based on the magnitude of these effects, respectively (**Table** **1**). Among the confirmed hits for SL1, compound P5 was notable for its significantly smaller size compared to the other binders and for containing bromine substituents, which may contribute to its target specificity (**Figure** **5**). However, P5 was not included in the ranking table, as its evaluation across all three experiments was hampered by severe LB upon SL1 addition, which prevented a reliable analysis of CSP and waterLOGSY. However, severe LB generally indicates intermediate exchange, consistent with moderate to tight affinity. WaterLOGSY factors were generally higher for PK hits (Table 1), likely reflecting differences in binding kinetics. PK binding occurs under fast exchange, enabling efficient magnetization transfer and stronger signals. In contrast, SL1 may bind under intermediate exchange, where LB‐related signal attenuation reduces the response despite potentially tighter binding.^[^ ^34^ ^,^ ^35^ ^]^ Differences also arise from RNA size, with distinct rational correlation times (≈4 ns for SL1 vs. ≈12 ns for PK). Next, we were interested in the structural basis for the observed selectivities of our validated hits. For RNA‐observed binding site mapping by NMR, we applied a focused selection strategy: 1) the best selective hit, defined as a compound that was both top‐ranked for one RNA target and experimentally confirmed not to bind the other, and 2) the strongest dual‐binder regardless of the predicted target.

**Table 1:** Ranking of hits identified in NMR screening experiments performed with the SARS‐CoV‐2 RNA elements SL1 and PK. In total, 77 compounds were screened against the targets SL1 and PK. For the quantification of the hits, three different NMR‐experiments were applied: 1) 1D‐^1^H, 2) waterLOGSY, and 3) *T*_*2*_‐relaxation experiments (*T*_*2*_ = 5 or 100 ms). The SL1 hit P5 was excluded from the ranking due to severe line‐broadening (LB) preventing correct evaluation. Compounds selected for further experiments are highlighted in lilac.

FBS rank	Hit of [RNA]	Compound	VS target [RNA]	CSP [Hz]	wLOGSY factor	*T*_*2*_ reduction [%]	FBS rank	Hit of [RNA]	Compound	VS target [RNA]	CSP [Hz]	wLOGSY factor	*T*_*2*_ reduction [%]
1	**SL1**	P33	PK	12.16	1.38	87.00	1	**PK**	**P80**	PK	9.68	2.52	96.00
2		P34	PK	11.32	1.09	94.00	2		S40	SL1	9.55	2.55	93.00
2		**P35**	PK	8.01	1.20	90.00	3		P33	PK	16.37	2.07	89.00
4		P82	PK	5.24	1.25	70.00	4		P34	PK	14.64	2.17	89.00
5		P64	PK	9.22	1.00	64.00	4		**P35**	PK	15.28	2.19	83.00
6		PS2	SL1 + PK	7.61	1.03	56.00	6		P81	PK	8.75	1.73	91.00
7		P81	PK	5.18	0.77	73.00	7		P22	PK	2.55	4.34	81.00
8		S40	SL1	5.28	0.00	43.00	8		P82	PK	8.77	1.56	88.00
8		P29	PK	4.43	0.89	49.00	9		P30	PK	4.50	2.19	66.00
10		**S14**	SL1	3.34	0.97	30.00	10		S1	SL1	5.27	2.89	22.00
							11		PS2	SL1 + PK	1.45	7.67	35.00
							12		S8	SL1	LB	4.11	42.00
							13		P32	PK	4.52	1.40	56.00
							13		P70	PK	6.51	1.88	20.64
							15		P31	PK	4.43	1.43	52.00
							16		P49	PK	0.00	1.36	48.00

This approach allowed us to prioritize compounds representing both selective and dual‐targeting compounds for more detailed structural characterization. Figure 4 highlights NMR screening experiments performed with the compounds S14 (SL1 selective), P80 (PK selective), and P35 (dual‐binder). Of the 77 compounds screened via NMR, 20 showed interaction with either one or both of PK and SL1, corresponding to a total hit rate of 26%. Out of these 20 hits, 11 interacted with SL1 and 16 with PK. Seven of these hits bound both target RNAs (dual‐binders). Thus, four compounds were selective for SL1 and nine for PK, respectively (Figure 5, Table 1). Initially, 44 compounds were computed to be selective for SL1, 88 for PK, and two were computed to interact with both targets. Thus, NMR could only partially validate this relatively high binding selectivity proposed by docking. PK had a higher number of hits, most likely due to its higher structural complexity and greater number of potential ligand‐binding sites compared to SL1, in line with the larger number of compounds computed to interact with PK. Most strikingly, a significant degree of selectivity swap between VS and NMR was observed, as reflected by eight hits for SL1 from the PK‐only library and three hits for PK from the SL1‐only library. In total, we identified one selective SL1 binder and seven selective PK binders that were consistent with the VS results. (Figure 5). Interestingly, only two compounds, PS1 and PS2, were originally predicted to interact with both RNAs in the VS. Of these, PS2 was indeed a confirmed hit for both RNAs.

### 2.3 Binding Affinities in the Low Micromolar Range for PK Align with NMR Screening Results and Indicate More Frequent, yet Less Selective, Ligand Interactions Compared to SL1

**Figure 6:**
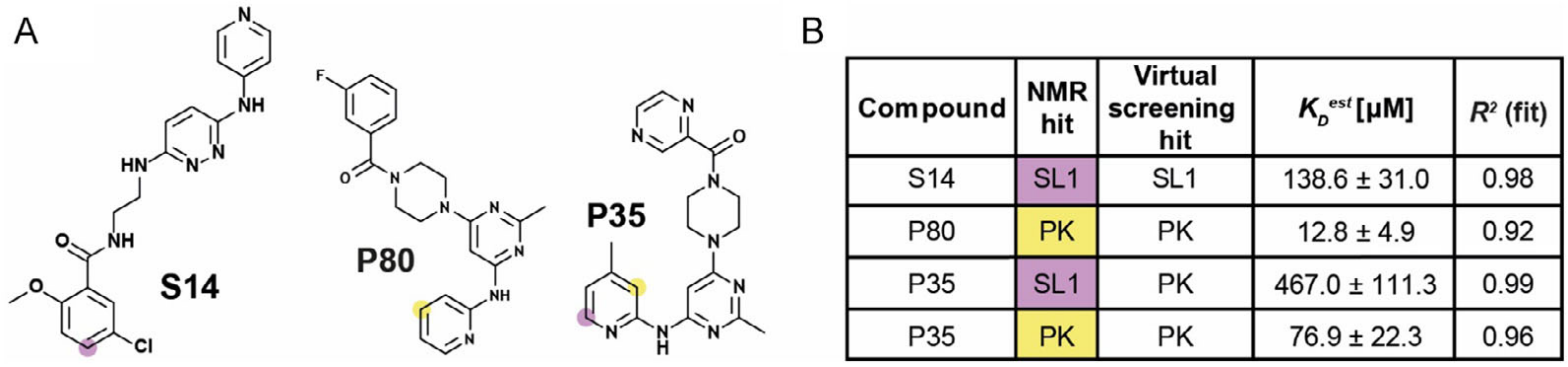
Ligand‐detected NMR titrations. Measurements of eight individual samples were evaluated based on shifts of the compounds induced by increasing concentrations of either SL1 or PK. A) We have selected one compound that has been shown to bind SL1 (S14) or PK (P80) individually, and one compound that has been shown to bind both (P35). Protons used for evaluation are highlighted in lilac (SL1) and yellow (PK). B) Estimated binding affinities of the NMR‐based ligand‐detected titrations.

We next performed NMR‐based titrations to estimate binding affinities. We selected a ligand each for SL1 and PK, respectively, as well as a compound that exhibited interactions with both viral RNA elements (**Figure** **6**A) that were positive in the VS and the NMR experiments. Interestingly, both compounds titrated with PK showed affinities in the low micromolar range, while those titrated with SL1 showed lower affinities (Figure 6B, Figure 4, Supporting Information). The PK‐selective compound P80 displayed the strongest interaction, with an estimated binding affinity *K*_*D*_^*est*^ ≈ 10 µM, while the SL1‐selective compound bound S14 showed a tenfold lower affinity. The dual‐binding compound P35 bound to PK, with *K*_*D*_^*est*^ ≈ 80 µM, which is weaker than that of the PK‐selective compound but stronger than its affinity for SL1 (≈sixfold lower affinity).

### 2.4 Binding Site Mapping via NMR Highlights the Potency of the Dual‐Binder P35, Inducing Strong CSPs both SL1 and PK RNA

Subsequently, samples were prepared for RNA‐detected NMR measurements to determine the binding site of ligands on the target RNA. All NMR experiments were performed with a fivefold excess of ligand to reach near saturation of RNA binding sites and to allow clear identification of binding‐induced spectral changes. We chose the following compounds to assess the structural basis of differential selectivity: P80 (VS and NMR Hit for PK), S14 (VS and NMR Hit for SL1), and P35 (VS and NMR dual‐target hit).

**Figure 7:**
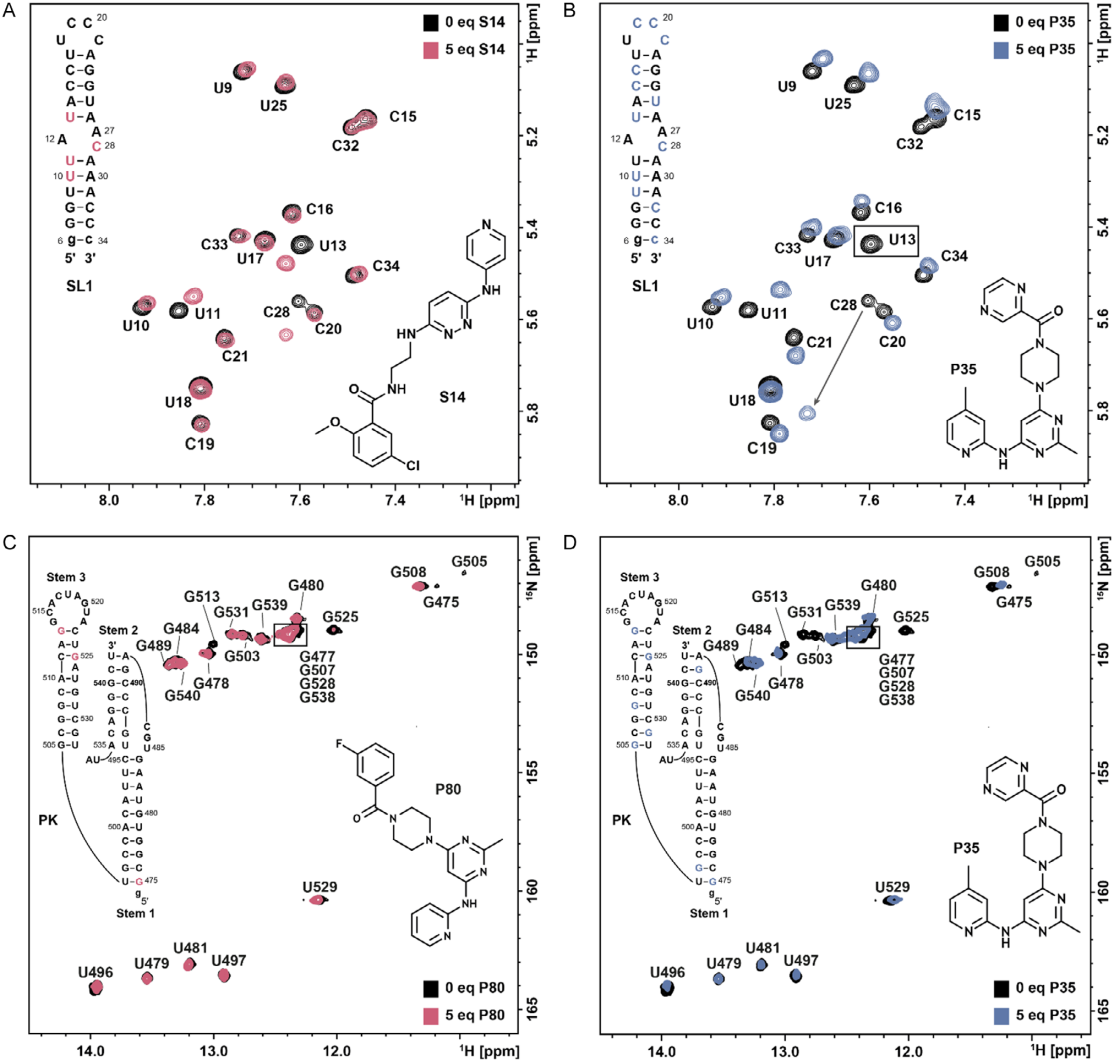
RNA‐detected NMR measurements were performed with the SARS‐CoV‐2‐derived RNA elements SL1 and PK in the presence of potential ligands. The measurements were conducted with one ligand identified as a hit for both viral RNAs, and an additional compound that demonstrated an interaction with only one of the viral RNA elements. A,B) 2D‐^1^H,^1^H‐TOCSY experiments recorded with SL1 in the absence (black) and presence of the compounds S14 (light burgundy) and P35 (blue). The black box shown in (B) highlights the severe LB of the residue U13. The arrow indicated the large shift of the residue C28. Nonisotopically labeled SL1 was used at a final concentration of 150 µM. C,D) 2D‐^1^H,^15^N‐TROSY experiments recorded with PK in the absence (black) and presence of the compounds P80 (light burgundy) and P35 (blue). ^15^N‐isotopically labeled PK was used at a final concentration of 150 µM.

**Figure 8:**
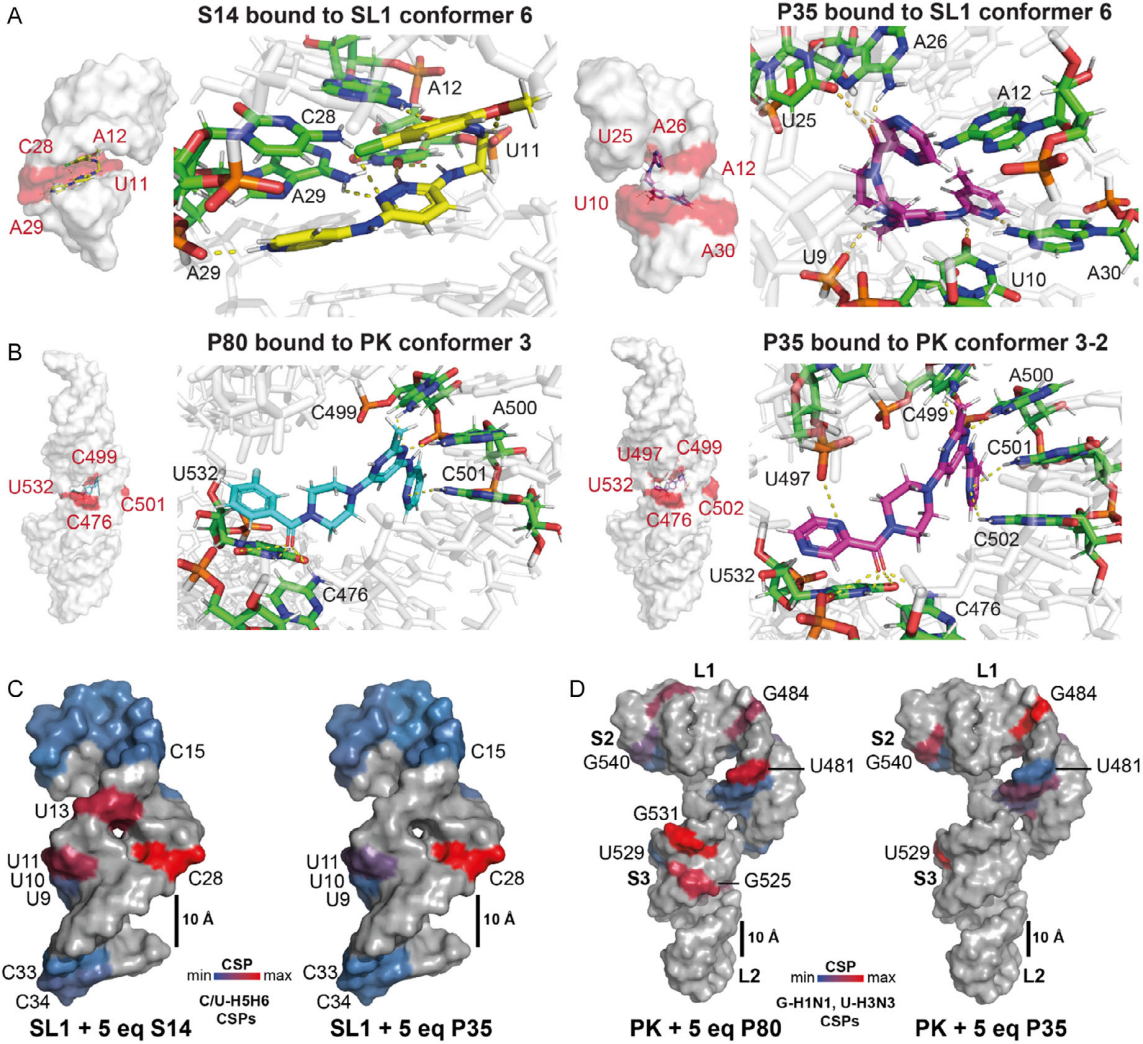
Mappings of calculated and experimental determined binding sites of the potential SARS‐CoV‐2 SL1 and PK ligands. A) Docking poses of SL1 and the compounds S14 and P35 highlighting potential H‐bond formations. B) Docking poses of PK and the compounds P80 and P35 highlighting the potential H‐bond formation. C) Pyrimidine H5H6 CSPs deriving from the 2D‐^1^H,^1^H‐TOCSY measurements were mapped onto the NMR‐determined 3D‐structure of SL1 (PDB entry: 9EOW).^[^ ^23^ ^]^ D) G‐H1N1 and U‐H3N3 imino group CSPs deriving from the 2D‐^1^H,^15^N‐TROSY measurements were mapped onto the adopted cryo‐electron microscopy 3D‐stucture of PK (PDB entry: 6XRZ).^[^ ^62^ ^]^ C,D) CSPs were normalized to the largest shift observed in each experiment and colored from low (blue) to high (red). Residues highlighted in gray were either not detected in the experiment or exhibited severe LB, which prohibited an exact analysis. Illustrated 3D‐coordinates of SL1 and PK do not correspond to their actual size difference (see scale bar). Calculated CSPs are shown the Table 2, Supporting Information.

For binding site mapping of SL1, we chose the 2D‐^1^H,^1^H‐TOCSY as it has proved successful in monitoring the chemical shifts of residues at and adjacent to the proposed binding site. For compounds S14 and P35, the internal loop of SL1 was the major binding epitope (**Figure** **7**A,B). For S14, SL1 residues U11, U13, and C28 exhibited major CSPs (**Figure** **8**C). These residues are located within or adjacent to the RNAs internal loop. Minor CSPs were detected for SL1's apical loop residues in the presence of both compounds, consistent with the predicted binding poses from docking (Figure 8A).^[^ ^23^ ^]^ For PK, we conducted 2D‐^1^H,^15^N TROSY experiments, as the TOCSY was not sufficiently sensitive (Figure 5, Supporting Information). For PK, the major effects were detected on the residues G475, G505, G513, and G525 in the presence of its binder P80 (Figure 7C,D). The NMR‐observed CSPs and LB effects of P80 to PK are in line with the predicted binding poses, in which P80 was placed in the same general location but had contacts to slightly different residues (Figure 8B,D). In our previous work, we demonstrated that these residues are implicated in potential ligand‐RNA interactions and thus, the CSP data reported here substantiated the hypothesis that the primary binding event occurs in the junction between stem 1 and stem 3 of the PK.^[^ ^29^ ^]^ Upon inspection of docking poses for S14 to SL1 and for P80 to PK, we found that in both cases every replicate with the best docking score bound in the same conformation with the same predicted hydrogen bonding patterns, indicating a strong preference for a single bound state (Figure 8). Additionally, we consistently observed S14 to make four predicted H‐bond contacts to SL1 while P80 made five or more predicted H‐bond contacts to PK, which could be a potential explanation for the difference in affinities.

Taken together, CSPs and LB effects upon ligand addition revealed distinct interaction patterns for both RNA elements. The selective binders, S14 and P80, induced localized perturbations consistent with binding at the predicted sites, respectively, supporting their selectivity for their respective target RNA (Figure 7A,C). In contrast, the dual‐binding compound P35 exhibited broad perturbations across both RNAs, indicating a flexible binding mode that could involve global changes in RNA conformation. (Figure 7B,D). These observations agree with the initial NMR screening results and support the predicted binding preferences derived from VS. Again, when looking at the predicted binding poses from docking, we saw a higher number of predicted H‐bonds from P35 to PK versus SL1, likely explains the observed difference in K_*D*_^*est*^ (Figure 8A,B).

### 2.5 SAR Analysis of Compounds Binding to SL1 and PK Revealed Chemical Features Associated with Selective Binding

To investigate SAR and potential scaffold‐based selectivity, we performed a comparative analysis of the ligands tested in this. study, using a combination of RDKit (for descriptor calculations and fingerprint analysis) and DataWarrior (for scaffold clustering and visualization).^[^ ^36^ ^,^ ^37^ ^]^ Compounds were categorized based on their binding behavior observed in NMR‐based screening: nonbinders, selective binders to either SL1 or PK, and dual‐binders.

We first focused on the physicochemical characterization of all measured compounds to identify potential correlations with binding behavior. Parameters, such as molecular weight (MolWt), lipophilicity (LogP), topological polar surface area (TPSA), number of hydrogen donors/acceptors, and number of rotatable bonds, were calculated and visualized via boxplots (Figure 6, Supporting Information). The statistical evaluation using Analysis of Variance (ANOVA) showed that MolWt and LogP differed significantly between the categories, with RNA‐binding ligands generally displaying higher values (Table 3, Supporting Information). This may reflect a tendency of binders to form multivalent or hydrophobic interactions with RNA structures. In contrast, descriptors like TPSA or hydrogen bonding capacity did not differ between the binding categories. We next applied Principal Component Analysis (PCA) and Uniform Manifold Approximation and Projection (UMAP) to explore patterns in descriptor combinations, but neither method showed clear separation between ligand categories, with the first two PCA components explaining ≈67.5% of the variance (Figure 7, Supporting Information). This indicates that while individual descriptors may be discriminative, their combined variation does not clearly distinguish binding behavior in low‐dimensional projections.

**Figure 9:**
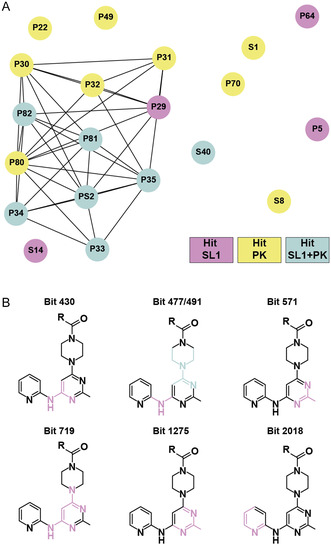
Structural comparison of compounds that interacted with the target RNAs validated via FBS**.** A) Tanimoto‐based similarity analysis of FBS hits was performed in DataWarrior using the SkelSpheres descriptor, independently of the experimental binding data. Circles represent individual compounds, and lines indicate structural similarity‐based connections between them. Lilac‐highlighted compounds interacted with SL1, yellow with PK, and blue with both RNAs in the FBS experiments. B) Substructures corresponding to significant Morgan fingerprint bits were found to discriminate between ligand categories. These bits were determined through bit‐level analysis using RDKit, where the frequency of occurrence of each of the 2048 bits was systematically compared across PK binder, SL1 binder and nonbinder. Corresponding substructures are highlighted in violet, except for bit 477, which is highlighted in blue.

The scaffold diversity was assessed using Tanimoto‐based clustering, allowing us to identify recurring core structures across binding profiles. First clustering only included the hits identified in the NMR‐based screening (**Figure** **9**A). Compounds such as P30‐P35, P81, and P82 form a dense cluster due to their shared aryl‐linked heterocyclic cores, while outliers like P5, S1, and S8 are structurally distinct and therefore appear disconnected. Scaffold cluster analysis revealed that all compounds but S40 that interacted with both RNAs in the NMR experiments are present in the major cluster, indicating a general RNA‐binding tendency of their shared scaffold. The nonselective binders frequently contained flexible linkers, polyaromatic systems, or amphiphilic motifs, potentially enabling them to engage a broader range of RNA architectures.

To visualize structural motifs associated with RNA binding, we performed a bit‐level analysis of Morgan fingerprints (Extended Connectivity Fingerprints, ECFP4) across the entire compound library, including binders and nonbinders. Frequently occurring bits in binders but rarely in nonbinders may be important for binding behavior. Detailed analysis showed seven such substructures in 36%–73% of the binders (Figure 9B, Table 4, Supporting Information). Bit 430 and 491 were the most common, occurring in 63% of PK and 73% of SL1 binders. All molecules with these bits are part of the main Tanimoto cluster, including S14. Similarly, bits 477 and 719 were highly prevalent, occurring in over 56% of the SL1 and PK binders (Figure 9B). This suggests that certain fragments generally contribute to RNA binding, but do not determine target specificity. However, bit 571 showed a different pattern, occurring more often in SL1 (64%) than PK binders (50%), and rarely (2%) in nonbinders. An additional methyl group on the pyrimidine scaffold may subtly influence binding preferences, suggesting a weak structural shift toward SL1 binding. Dimensionality reduction techniques tested were not conclusive. PCA and UMAP plots reflect only a small part of the actual differences between the molecules, and 32 components are required to fully explain variance (Figure 8). Therefore, they were not included. Fingerprint analysis focused directly on the presence and absence of specific sub‐structural features to better understand RNA binding.

### 2.6 Integration of Docking and NMR shows that Conformer‐Dependent Binding Drives Selectivity in SL1 and PK, while Overall Binding Regions are Accurately Predicted

Docking of the dual‐binder P35 to PK revealed comparable affinities across several conformers, including 3–2, which is consistent with its broader binding profile (Figure 9A, Supporting Information). In contrast, P80, P81, and P82, as well as other selective PK binders, showed a clear preference for conformer 3–2. This suggests that, while this conformation contributes to affinity, it does not determine selectivity alone (Figure 9B, Supporting Information). These observations are consistent with the NMR data, in which P80 induced strong, specific CSPs, whereas P35 caused broader perturbations. Therefore, while residue‐level interactions could not be precisely predicted, the binding region and overall shape complementarity were correctly identified through docking.

**Figure 10:**
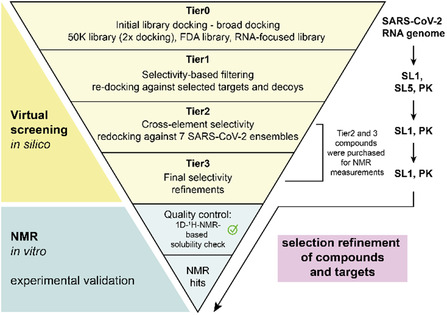
Summarized workflow highlighting the filtering potential targets and compounds via virtual screening and NMR. Starting with compounds from three libraries, Tier0 involved broad docking to seven SARS‐CoV‐2 RNA ensembles. Compounds passing statistical cutoffs for three targets (PK, SL1, SL5) advanced to Tier1 for selectivity screening against HIV TAR and RRE decoys. Tier2 applied cross‐target selectivity filtering; final selectivity refinements led to Tier3. Tier3 plus selected Tier2 hits were tested by NMR (QC included). This approach enabled reliable identification of RNA‐targeting compounds.

For SL1, a more structurally simple helix‐junction‐helix RNA, the pattern was different. In this case, there was little difference in the scores across conformers for either the selective or nonselective hits (Figure 10, Supporting Information). Rather, certain conformers showed more favorable scores in general across the hits, such as conformers 6 and 10, but this was not consistent among all hits. It is less clear what is driving selectivity for SL1, a possibility is that certain residues are important for selective H‐bonding contacts. For SL1, docking was more accurate at determining the exact residues contacted by P35 and S14 that we observed contacts with experimentally, though this increase in accuracy is likely due to the smaller size and search space for the SL1 conformers. As with the NMR studies, docking predicted that most binding occurs in the bulge region, with some activity in the loop as well.

## 3 Conclusion

In this study, we demonstrated the utility of a combined virtual screening and NMR‐based approach to identify small‐molecule binders of two conserved SARS‐CoV‐2 RNA structural elements, SL1 and PK. Our computational filtering strategy enabled the prioritization of a broad pool of candidate compounds and highlighted the druggability of SL1 and PK. Binding affinities in the 10 µM regime for ligands binding to the RNA elements could be achieved which compares well with more elaborate medicinal chemistry efforts. Within this approach, the choice of the two RNA targets proven successful. In direct comparison, the other RNA elements did not progress beyond Tier1, and reaching Tier1 was also only the case for one RNA element. The performed multireplicate docking approach evaded inherent docking biases, such as limitations in conformational sampling and RNA as target itself and applied stringent score‐based filtering to reduce the large initial chemical space (**Figure** **10**).^[^ ^38^ ^,^ ^39^ ^]^ The selection of compounds that can discriminate between different viral RNA elements was achieved through selectivity‐focused cross‐docking against HIV‐derived RNA decoys in Tier1 and Tier2, which predicted a small degree of selectivity. This procedure led to a refinement of a compound list further used in NMR‐based experiments for in vitro validation of binding interactions shown in the virtual screening. However, the significantly higher number of predicted hits compared to experimental validation highlights the limitations and potential bias inherent in current RNA docking algorithms and force fields. It further revealed that the selectivity predicted in Tier3 docking was not confirmed experimentally. Several compounds that exhibited strong computational selectivity for PK or SL1 displayed similar binding characteristics or cross‐reactivity in solution. There was no clear correlation between Tier3 exclusivity and NMR‐detected specificity. These results imply that, although the final Tier3 docking step is conceptually valuable, it may not offer additional benefits beyond Tier2 for evaluating compound selectivity for structurally diverse RNA targets.

Overall, while docking remains an essential tool for early hit identification, our findings highlight the importance of integrating experimental methods early in the screening pipeline. The collected NMR‐based results underscore the value of integrating RNA‐detected NMR techniques with in silico screening to dissect the molecular recognition properties of small molecules targeting structured RNAs. Combined with binding affinities determined by NMR, the binding site mapping supports the conclusion that the analyzed compounds bind in the intermediate to fast exchange regime. Bit‐level fingerprint analysis identified several sub‐structural motifs that were highly enriched in both PK and SL1 binders but largely absent in nonbinders. However, a clear structural rationale for selectivity between PK and SL1 remains elusive, possibly due to overlapping recognition elements, reflecting the diverse conformational landscapes and binding flexibility of viral RNA elements. The relatively small number of active compounds and targets further limits firm conclusions about selectivity. Although VS and NMR hits do not overlap perfectly, combining the two approaches effectively expands the pool of promising RNA binders. Computational screening prioritizes candidates from a large chemical space, and NMR provides experimental validation, which makes this pipeline valuable. Nevertheless, the identified motifs provide a useful foundation for future rational ligand design and underline the broader relevance of integrative structural biology approaches, in which computational modeling, biophysical measurements, and structural analysis converge to improve our understanding of RNA‐ligand recognition. Although the highlighted compounds bind SL1 and PK with low micromolar affinities, their selectivity over human RNAs remains an open question, and addressing potential off‐target interactions presents the next step toward validating their specificity and therapeutic relevance. Overall, our work advances the framework for rational RNA‐targeted drug discovery and highlights both the opportunities and challenges in targeting viral RNA with high specificity.

At the beginning of the project, experimentally determined NMR structures of SARS‐CoV‐2 RNAs were still being developed and were not publicly available. Therefore, our workflow depended on modeled ensembles. The accessibility of experimental NMR RNA structural ensembles is, however, progressing rapidly and thus, integrating emerging NMR structures can further refine virtual screening and improve selectivity predictions.^[^ ^23^ ^,^ ^40^, ^41^, ^42^, ^43^, ^44^ ^]^ This approach is supported by recent work by Arhin and Keane who applied a similar structure‐guided methodology using NMR‐derived RNA ensembles to identify small‐molecule ligands targeting the oncomiR‐1 NPSL2 hairpin.^[^ ^45^ ^]^ Together, these studies underscore the potential of integrating emerging structural data with computational and biophysical methods to accelerate the discovery of drugs targeting RNA. They also demonstrate the broader applicability of these integrative strategies to diverse RNA targets.^[^ ^46^, ^47^, ^48^, ^49^, ^50^, ^51^, ^52^, ^53^, ^54^, ^55^, ^56^, ^57^, ^58^, ^59^, ^60^, ^61^, ^62^ ^]^

## Supporting Information

The authors have cited additional references within the Supporting Information.

## Conflict of Interest

The authors declare no conflict of interest.

## Author Contributions

**Sabrina Toews**: conceptualization (lead); data curation (lead); formal analysis (lead); investigation (lead); validation (lead); writing—original draft (lead); writing—review & editing (lead). **Betül Ceylan**: conceptualization (lead); data curation (lead); formal analysis (lead); investigation (lead); methodology (equal); writing—original draft (supporting); writing review & editing (supporting). **Anna Wacker**: conceptualization (lead); funding acquisition (supporting); project administration (equal); supervision (equal); writing—original draft (lead); writing review & editing (lead). **Megan Ken**: conceptualization (lead); data curation (lead); formal analysis (lead); funding acquisition (lead); investigation (lead); methodology (lead); writing—original draft (equal); writing—review & editing (lead). **Harald Schwalbe**: conceptualization (lead); funding acquisition (lead); project administration (lead); supervision (lead); writing—original draft (equal); writing—review & editing (lead). **Sabrina Toews** and **Betül Ceylan** shared co‐first authorship.

## Supporting information

Supplementary Material

## Data Availability

Raw data are available at https://doi.org/10.25716/gude.1hf6‐w87q (Goethe University Data Repository, GUDe). The data that support the findings of this study are available from the corresponding author upon reasonable request.
