# Supplementary material for: Integrative In Silico and In Vitro Screening of Low Molecular Weight Compounds Targeting SARS‐CoV‐2 RNA Elements
Source: Chembiochem. 2025 Nov 29;26(24):e202500668. doi: 10.1002/cbic.202500668 (PMC12703450; doi:10.1002/cbic.202500668)
Supplement: Supplementary file 1 — Supplementary Material [file CBIC-26-e202500668-s001.zip › cbic70159-sup-0001-SuppData-S1.pdf]

Supplementary Information

Integrative *In Silico* and *In Vitro* Screening of Low Molecular Weight Compounds Targeting SARS-CoV-2 RNA elements

Sabrina Toews,[a,b]‡ Betül Ceylan,[a,b]‡ Anna Wacker,[a,b] Megan Ken,*[c] and Harald Schwalbe*[a,b]

[a, b] Sabrina Toews, Betül Ceylan, Dr. Anna Wacker, Prof. Dr. Harald Schwalbe
[a] Institute for Organic Chemistry and Chemical Biology, Goethe University Frankfurt, Frankfurt am Main, Germany
Max-von-Laue-Strasse 7, 60438 Frankfurt am Main (Germany)
[b] Center of Biomolecular Magnetic Resonance (BMRZ), Goethe University Frankfurt, Frankfurt am Main, Germany
Max-von-Laue-Strasse 9, 60438 Frankfurt am Main (Germany)
E-mail: schwalbe@nmr-uni-frankfurt.de

[c] MD, Dr. Megan Ken
The Scripps Research Institute
Department of Integrative Structural and Computational Biology
La Jolla, CA, USA
E-mail: mken@scripps.edu,

‡ Shared co-first authorship

* corresponding authors

Material and Methods

Computational Docking

Receptor Preparation

The docking software Internal Coordinate Mechanics (ICM) from Molsoft Inc. was used to prepare the RNA receptors and dock the small molecule library. RNA ensembles for the seven elements docked (5'-UTR: SL1, SL2, SL4, SL5; PK; 3'-UTR: SL1 and SL2) were reported in Rangan *et al.* and the structure files were retrieved from https://github.com/DasLab/FARFAR2-SARS-CoV-2.[10] The 10 best scoring conformers for each element were used as the ensemble. Each conformer was loaded into ICM and converted to an ICM object. The PocketFinder function was used to identify druggable pockets and define the receptor around them as previously performed by Ganser *et al*.[13, 30] These pockets and their receptors are shown in SI Figure 1. For most conformers there was one pocket determined, for conformers with no pocket defined on the default setting that conformer was removed. For conformers with multiple pockets defined, multiple receptors were made for that conformer.

Library Preparation

The 50K diversity and RNA-focused libraries were downloaded from Life Chemicals Inc. at https://lifechemicals.com/. The FDA-approved compound library was downloaded from the ZINC 15 database using the "fda-only" filter.[33] To account for potential local pH differences that may occur experimentally, each ligand had three protonation states calculated, pH = 5.4, 7.4, and 9.4. This was done using ChemAxon software (https://chemaxon.com/). Any duplicate states were then removed using the "unique-only" function with OpenBabel software (https://openbabel.org/). The compounds then had their energy minimized using ChemAxon software and any bond length errors were corrected. The libraries were converted to ICM format for docking. The default option to protonate the ligands at physiological pH was turned off.

Initial Docking – Tier0

Tier0 indicates the docking scores for the full libraries. In this step, the 50K library was docked twice against each ensemble, and the FDA and RNA-focused libraries were docked five times against each ensemble (except for the 3'-UTR elements SL1 and SL2, in which the FDA and RNA-focused libraries were each docked twice). Because each RNA ensemble has multiple receptors, every docked molecule has a score for each receptor. The molecule's ensemble docking score is calculated by averaging the scores for each receptor within the ensemble. We averaged the scores two ways; an arithmetic average (AA) in which the sum of receptor scores is divided by the number of receptors, and a Boltzmann average (BA) in which the scores are weighted as described in the following equation (k_B = Boltzmann constant, T = temperature):

$$[1] \qquad \langle BA \rangle = \frac{\sum_i A e^{-E_i/k_B T}}{\sum_i e^{-E_i/k_B T}}$$

This was done for each docking replicate (either two or five replicates as described above), and then the top score of all replicates was taken as the final score to use for the next filtering stage. The scores resulting from both methods of averaging are contained in the **Supplementary_List_A**. The score distributions are shown in SI Figure 2. We applied a filter to the Tier0 docking results to determine our hits for the next stage of virtual screening, Tier1. The filter was applied to both the AA and BA datasets, and any duplicates between the two were eliminated. For both datasets the score filter was set at three standard deviations below the mean of the full library scores, excluding any scores greater than zero. For the BA datasets, an additional cutoff score of -35 was applied. This number was determined based on prior work in the lab showing that true hits rarely score higher than -35. The values of these cutoffs and the number of compounds that passed from each library for each ensemble is listed in the **Supplementary_List_B**. Due to the amount of docking and downstream processing in subsequent steps, we used the data from Tier0 analysis to choose only three elements to move forward with. This decision was based on potential relevance of the RNA element as a drug target as well as the number of compounds that passed the Tier0 cutoffs, weighing the performance of the 50K diversity library more

heavily as it is more likely to contain unique, selective chemical scaffolds. With these criteria, we moved forward with SL1, SL5 (5'-UTR), and PK.

Selectivity Cross-Docking – Tier1/Tier2

This next docking step is designed to virtually screen for selectivity. The compounds that passed the filters in Tier0 were then redocked against their respective RNA element ensembles with ten replicates and then docked against two decoy RNA ensembles with five replicates. These decoy RNA ensembles are both HIV RNAs with fully experimentally determined ensembles. They are the trans-activation response element (TAR) and the Rev-response element (RRE). Our assumption is that compounds with similar docking scores to their intended SARS-CoV-2 element as to the decoy RNAs are more likely to be nonspecific when tested experimentally. The scores for all Tier1 libraries against their targets and the decoys are listed in the **Supplementary_List_C**. Once again, we calculated the scores with both arithmetic average and Boltzmann average for each replicate. To increase our chances of discovering a selective binder, we implemented the following cutoff to the Tier1 docking scores: A compound's mean score (calculated as the sum of all replicate scores divided by the total number of replicates) to its target SARS-CoV-2 RNA ensemble must be lower than three standard deviations below its mean score to both decoy RNA ensembles. The filtering analysis showing these mean and cutoff scores, along with the designation of passing to Tier2 or not, is in the **Supplementary_List_D**. Visualization of the relative docking scores for compounds that passed to Tier 2 is shown in SI Figure 3.

Selectivity Refinements – Tier2/Tier3

Surprisingly, SL5 had almost no compounds pass the selectivity filter, so we went forward with SL1 and PK, both of which had >50 compounds pass the selectivity filter for the 50K library. SL1 and PK have both been shown to have potential biologically relevance as a drug target, and have two very different structures, making them experimentally interesting to compare. We surveyed the Tier2 hits for SL1 and PK to determine if further docking was required before selecting compounds for purchase. In terms of the FDA library, SL1 had no hits pass the selectivity filter. PK had three hits pass, but on visual inspection of docked poses they did not look like promising candidates, so we decided to not move forward with any FDA-approved compounds in our physical screening assay. For the RNA-focused library, SL1 had five compounds pass the selectivity filter and PK had six, we moved all to the list to purchase with no further investigation. The 50K library had 48 unique hits pass the selectivity filter for SL1, and 63 for PK. With these 111 compounds, we wanted to do one more computational selection experiment to make predictions about which compounds would be the most selective when experimentally testing, and potentially to help decide which compounds to purchase. For this final step, we performed four replicates redocking all the SL1 and PK Tier2 compounds against all seven SARS-CoV-2 ensembles and then looked at selectivity across RNA elements. The scores for this final docking are in the **Supplementary_List_E**. The comparisons across elements are shown in the **Supplementary_List_F**. For the PK element, 30 compounds showed selectivity against PK alone across all elements and thus moved into Tier3, and for SL1 8 compounds moved to Tier3. We then purchased as many compounds in Tier3 as possible given availability and cost, as well as some in Tier2 to increase our chance of finding true hits and test for the accuracy of cross-docking for selectivity. The list of compounds purchased is shown in Supplementary Table 1.

RNA synthesis

RNA samples were prepared following the method described by Wacker, Weigand *et al.*[2] For in-house synthesis, previously amplified and linearized double-stranded template DNA was incubated at 37 °C for 6 hours with the bacteriophage-derived RNA polymerase T7, along with cofactors and substrates (nucleoside triphosphates (NTPs), Mg^{2+}, dithiothreitol, spermidine) in a buffer containing 0.2 M Tris-HCl pH 8.0.[47,48] When NMR experiments required isotopically labeled RNA, the transcription included labeled NTPs. Thus, ^{15}N-labeled NTPs were used to enable the measurement of 2D-^{1}H,^{15}N-TROSYs. The *in vitro* transcribed RNA was purified using denaturing polyacrylamide gel electrophoresis, followed by further purification with reverse-phase (RP) high-performance liquid chromatography (HPLC). The final RNA samples were buffer-exchanged to 25 mM potassium phosphate (KP_i) and 50 mM potassium chloride (KCl) using 3-kDa molecular weight cut-off (MWCO) VivaSpin filtration units (Sartorius).

NMR experiments

NMR measurements were carried out using a Bruker 600 AV NEO spectrometer with different cryo probes for 1.7 mm/3 mm tubes. Data were processed and analyzed using TOPSPIN 4.4.0 (Bruker BioSpin, Germany). The spectrometer was equipped with a SampleJet (Bruker BioSpin, Germany) for automated measurements performed with the IconNMR software (Bruker BioSpin, Germany). Samples were prepared in either 1.7 mm or 3 mm NMR tubes, which were placed in NMR tube racks that are compatible with the SampleJet.

1D-NMR experiments for fragment-based screening

The experiments were performed and evaluated as previously shown by Sreeramulu, Richter *et al.*[29]

1D-^{1}H

1D-^{1}H experiments were recorded either with excitation sculpting with gradients or jump return echo water suppression pulse sequences.

waterLOGSY on ^{1}H

waterLOGSY is employed to assess ligand affinities for macromolecules such as RNA and proteins and is frequently used in fragment-based hit discovery.[43] When RNA and a ligand are measured together and binding occurs, positive signals in the spectrum indicate a bound fragment, whereas negative signals indicate an unbound ligand. In the screening experiments conducted, water signals were suppressed using the solvent-optimized double gradient spectroscopy (SOGGY) sequence.[49]

T_2-CPMG on ^{1}H

Carr-Purcell-Meiboom-Gill (CPMG) experiments determine the T_2 relaxation time using a spin-echo pulse sequence.[50] Measured T_2-relaxation strongly depends on the rotation correlation time of the observed molecule. When ligand and RNA are measured together and binding occurs, T_2-relaxation rates change in comparison with samples only containing RNA or the ligand of interest. T_2-relaxation decreases because the larger rotational correlation times of the RNA-ligand complex causes signal broadening, unlike the sharper signals observed for the free ligand. Water signals were suppressed using the SOGGY sequence.[49]

2D-NMR experiments for binding side mapping

2D-^{1}H,^{1}H-TOCSY

Total correlation spectroscopy (TOCSY) experiments are utilized to observe cross peaks not only between directly coupled nuclei but also between nuclei connected through a series of couplings via Homonuclear Hartmann-Hahn transfer.[51,52] This experimental setup facilitates the identification of intra-nucleobase pyrimidine H5H6 cross peaks. In these experiments, water suppression is accomplished by using an excitation sculpting pulse sequence in the direct dimension. H5H6 CSPs were calculated as Euclidean distances using equation [2]:

$$[2] \qquad CSP\ [\Delta\delta_{HH}] = \sqrt{(\Delta\delta_{H5})^2 + (\Delta\delta_{H6})^2}$$

2D-^{1}H,^{15}N-TROSY

Two-dimensional transverse relaxation-optimized spectroscopy (TROSY) experiments employing both the proton (^{1}H) and nitrogen (^{15}N) nuclei are used to enhance the detection of ^{15}N-attached protons in large or flexible RNA molecules by minimizing transverse relaxation effects and improving spectral resolution.[53,54] This experiment enables the observation of ^{1}H,^{15}N correlations, providing site-specific information on imino and amino groups within nucleobases. This information is crucial for characterizing RNA secondary and tertiary structures. Water suppression is accomplished by using pulsed field gradients and selective excitation sculpting, ensuring clear detection of labile protons involved in hydrogen bonding and base pairing. G-H1N1 and U-H3N3 CSPs were calculated as Euclidian distances using equation [3]:

$$[3] \qquad CSP\ [\Delta\delta_{HN}] = \sqrt{(\Delta\delta_H)^2 + (0.1\Delta\delta_N)^2}$$

NMR-based approach for the estimation of binding affinities

Binding affinities of the fragments were determined via NMR as previously described by Sreeramulu, Richter *et al.*[29] The fragment concentrations remained constant (100 µM) while the RNA concentration was progressively increased (0 to 250 µM). Eight distinct samples were prepared in 1.7 mm or 3 mm NMR tubes, each with a total volume of 40 µL or 200 µL, respectively. The samples were buffered with 25 mM KP_i (pH 6.2), 50 mM KCl, and 5% deuterated dimethyl sulfoxide (DMSO-d_6) as the lock solvent and reference. The final 1D-^{1}H NMR experiments were performed at 298 K and 600 MHz, utilizing excitation sculpting for solvent suppression. Changes in chemical shift perturbations (CSPs) were analyzed and used to determine binding affinities through a non-linear fit equation assuming a single site-specific binding (Equation [4]). The binding affinity is regarded as *estimated dissociation constant* (K_D^{est}) as concentration-dependent CSPs often do not reach saturation.[55] In this equation, *L* and *R* denote the compound and RNA concentrations in µM, respectively. $\Delta\delta_{max}$ indicates the highest chemical shift detected in the titration series.

$$[4] \qquad \Delta\delta_{obs} = \Delta\delta_{max}\left\{\frac{([L]_0 + [R]_0 + K_D{}^{est}) - \sqrt{([L]_0 + [R]_0 + K_D{}^{est})^2 - 4[L]_0[R]_0}}{2[L]_0}\right\}$$

Molecular characterization and structural data analysis

All data processing, analysis and visualization steps were carried out in Phython using RDKit (v2024.03.5. *Open-source cheminformatics. https://www.rdkit.org*) within a Jupyter Notebook (v7.0.8.), running on Anaconda distribution (v24.5.0, *Anaconda Software Distribution. Computer software. https://anaconda.com*).[56] Pandas (v2.2.2.) library was used for data processing and data management.[57] Statistical evaluations were carried out using the spicy.stats module of the SciPy library (v1.14.0.).[50] For dimensionality reduction techniques, Principal Component Analysis (PCA) was performed with scikit-learn (v1.6.1.), and Uniform Manifold Approximation and Projection was carried out using the umap-learn package (v0.5.7). Matplotlib (v3.9.1.) and Seaborn (v0.13.2.) were used for creating charts and heatmaps.[58-62] Via NMR-measured compounds were categorized according to their observed binding behavior in NMR-based screening experiments. The classification included four groups: selective binders to SL1 (SL1 binder) and PK (PK binder), dual-binders interacting with both RNA targets (dual-binder) and molecules that showed no interaction (non-binder). To consider the influence of 3D molecular structure, ligands were prepared following a standard protocol (*Getting Started with the RDKit in Python. https://www.rdkit.org/docs/GettingStartedInPython.html*). Starting from the 2D representations, explicit hydrogen atoms were added. For each compound, up to 200 conformers were generated, followed by energy minimization using MMFF94s force field. For each molecule, key physicochemical descriptors were calculated to quantify molecular properties relevant to RNA recognition. These included molecular weight (MolWt), lipophilicity (LogP), topological polar surface area (TPSA), number of hydrogen bond donors and acceptors (NumHDonors, NumHAcceptors), and the number of rotatable bonds (NumRotatableBonds). To statistically assess descriptor differences between ligand categories, one-way analyses of variance (ANOVA) tests were applied. Statistical

significance was defined as a p value of < 0.05. For detailed structural characterization, Morgan fingerprints (Extended Connectivity Fingerprints, ECFP4) were generated for all ligands using a radius of 2 and a fingerprint length of 2048 bits. Radius of 2 captures chemical environments up to two bonds around each atom. To identify sub-structural patterns that may contribute to RNA binding, a bit-level analysis of these fingerprints was performed. Fingerprint bits were considered potentially discriminative if they occurred in more than 50 % of compound within at least one binding category (either PK or SL1 binders), while being present in fewer than 10 % of the non-binding compounds or entirely absent. We used DataWarrior (v06.04.02) to perform chemical structure similarity analysis on a dataset of 20 binder.[36] For structure comparison, we used the SkelSpheres descriptor, which captures topological features of molecular scaffolds. Pairwise similarity scores were calculated using the Tanimoto coefficient and compounds were grouped using hierarchical clustering based on these scores.

Supporting Figures

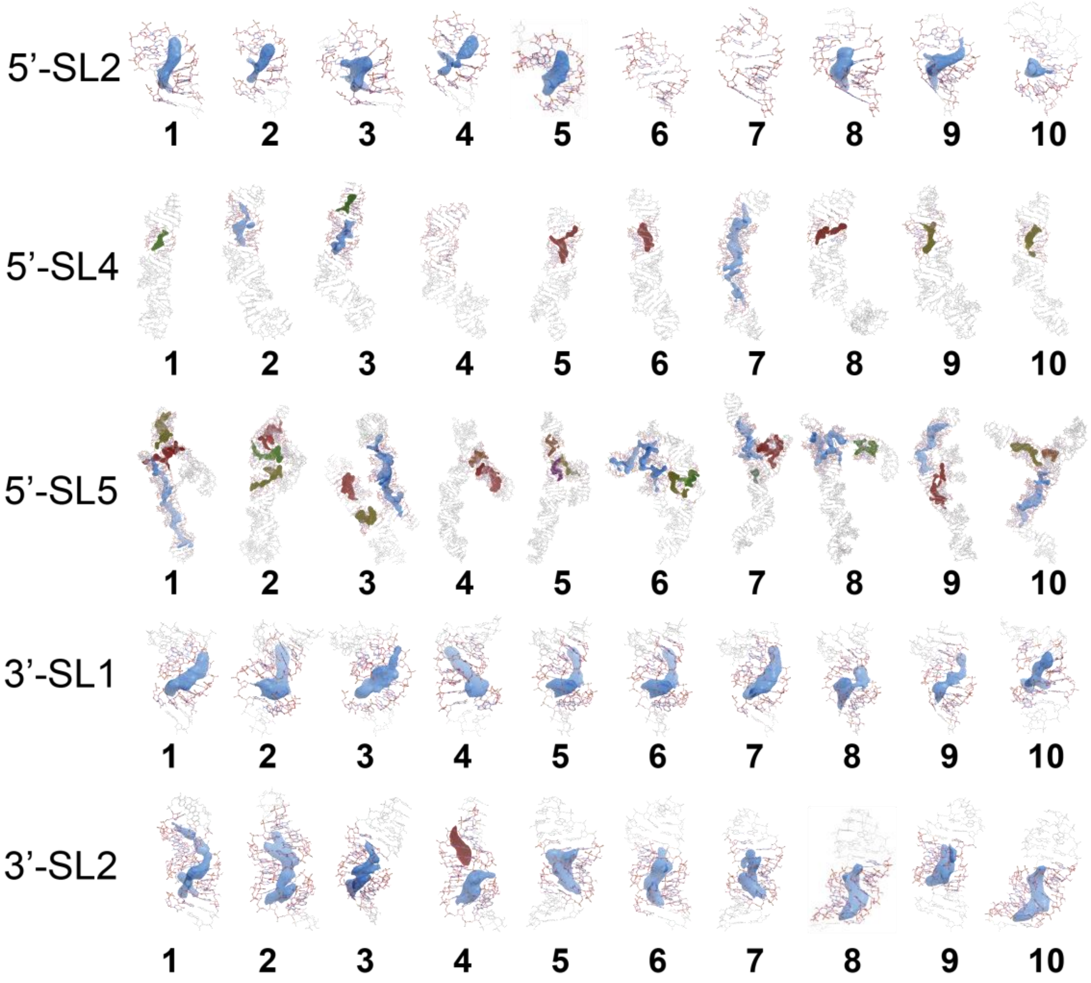

Supplementary Figure 1. Structures of the RNA ensemble conformers and the pockets used for receptor generation. For each ensemble we used the top 10 most energetically favorable conformers and then used the ICM Pocketfinder function to determine the most favorable location for small molecule binding.[30] Pockets were defined to be residues within 5 angstroms of the colored region(s), and these selected residues are shown in color with the rest of the RNA in gray. For conformers in which multiple pockets were identified, multiple receptors were created. For conformers in which no pockets were identified, a relevant region of the RNA was selected.

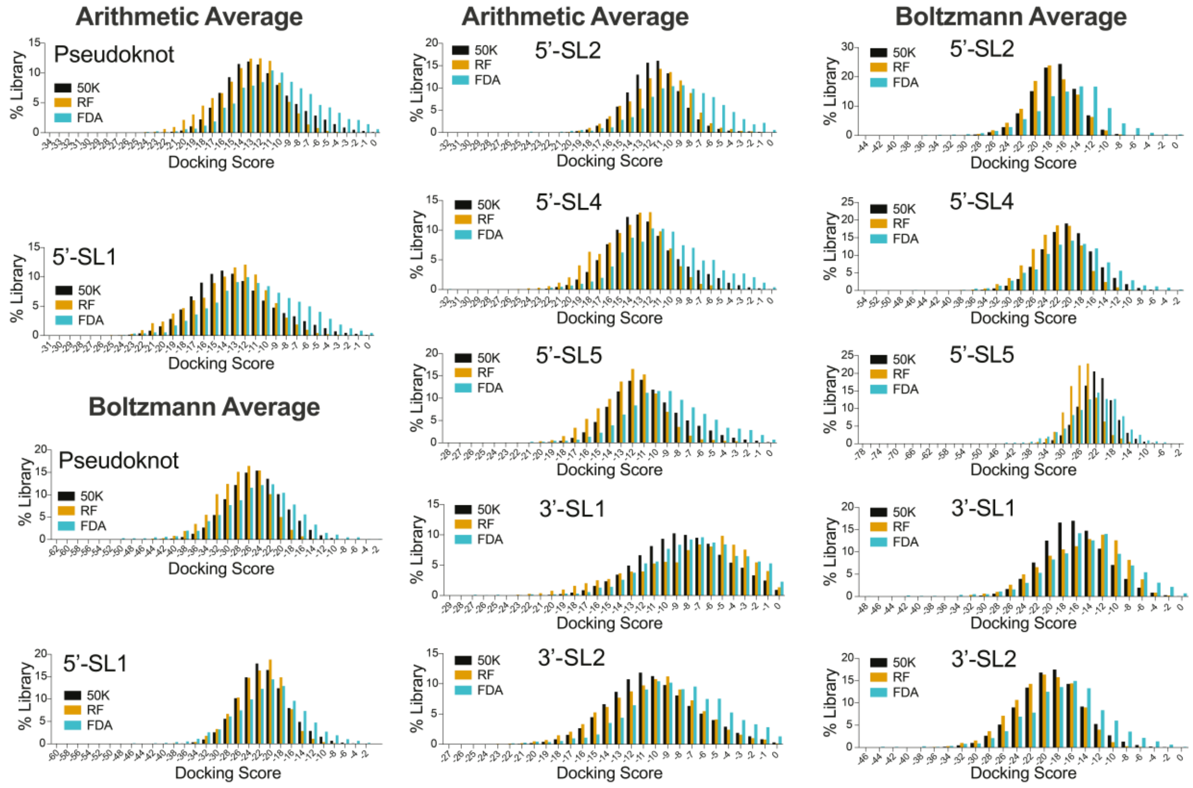

Supplementary Figure 2. **Tier0 docking score distributions and hit selection.** Compound libraries were docked against RNA ensembles with scores averaged using arithmetic (AA) or Boltzmann (BA) methods. The best score across replicates was retained. Hits were selected using a threshold of three standard deviations below the mean (excluding positive scores); BA datasets also required scores ≤ -35. Duplicates were removed.

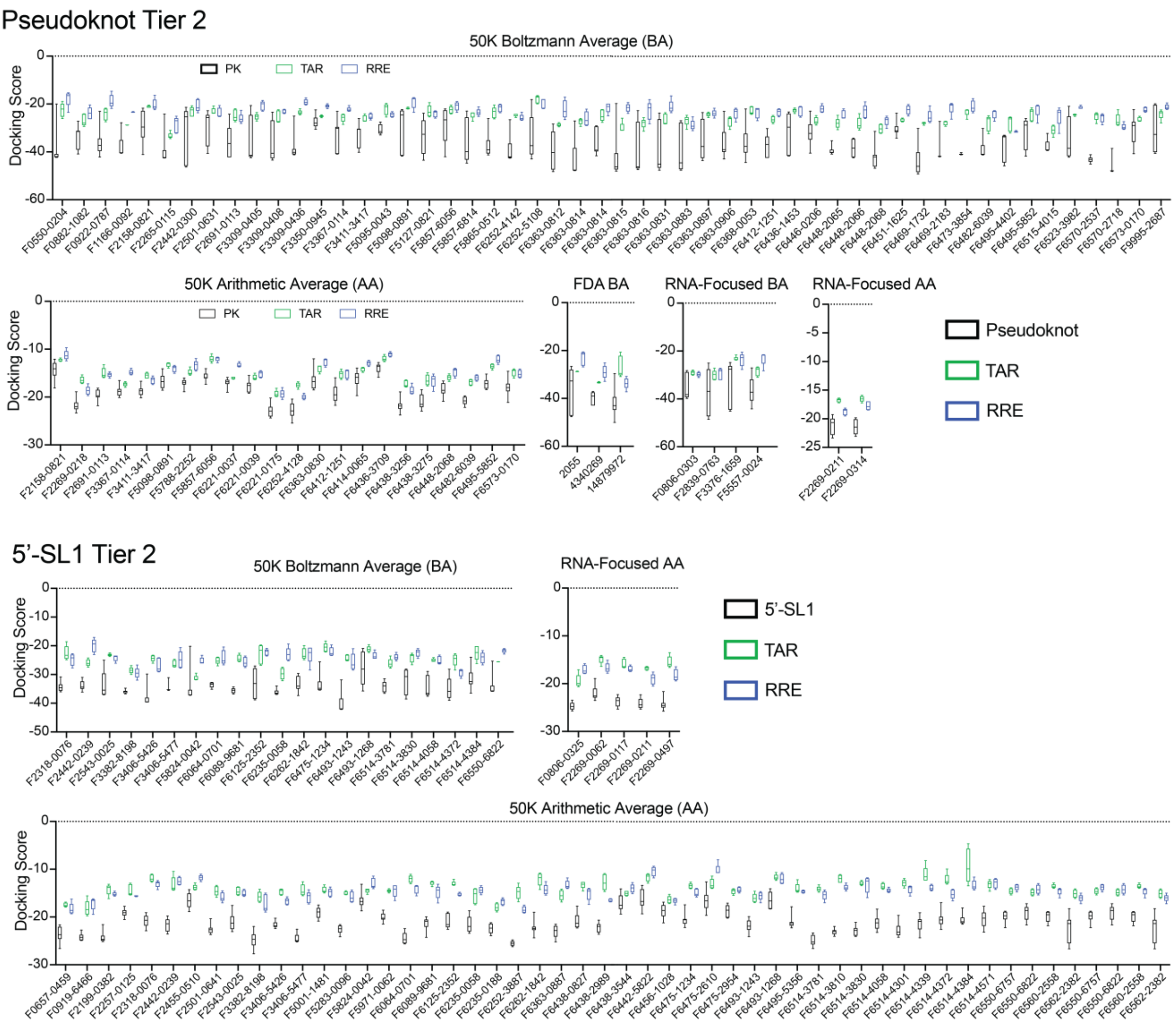

Supplementary Figure 3. Hits from Tier1 screening that passed to Tier2 – SL1 and PK. In Tier1 we docked compounds 10x against their target RNAs (black) as well as two decoy RNA ensembles, HIV TAR (green) and RRE (blue). All compounds shown here passed the selectivity screen, meaning the average score against the target is greater than 3 standard deviations below the mean of both decoy ensembles.

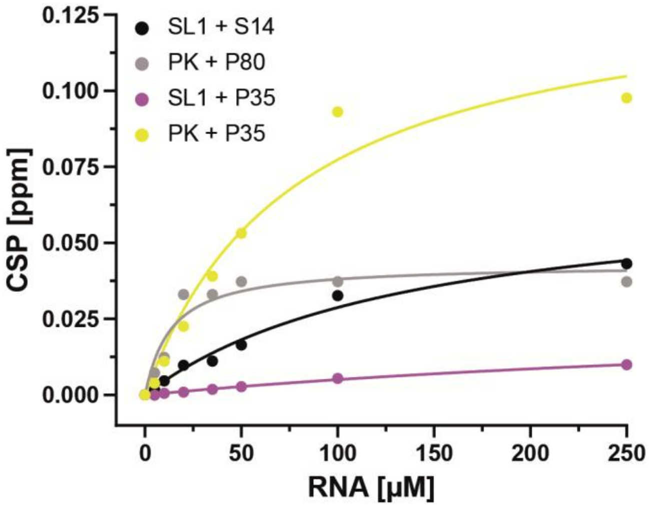

Supplementary Figure 4. **Non-linear fitting of ligand-detected titrations performed by NMR.** Non-linear fitting of the determined CSPs was performed for the estimation of the binding affinities K_D^{est} (SI equation [4]). The binding affinities were determined for a selection of compounds, which had previously been classified as hits in the preceding FBS experiments.

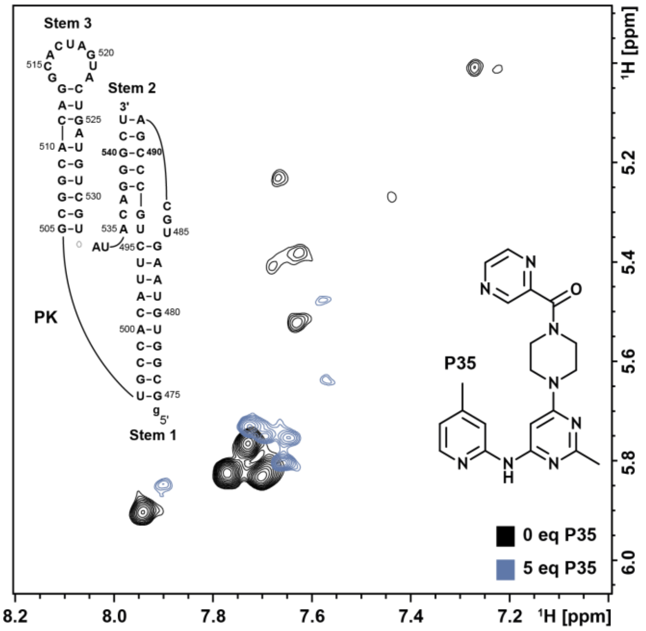

Supplementary Figure 5. **2D-^{1}H,^{1}H-TOCSY experiments recorded with PK in the absence (black) and presence of the compound P35 (blue).** Non-isotopically PK was used at a final concentration of 150 µM. Measurements were performed in the absence and 5-fold excess of ligand at a final DMSO-d_6 concentration of 5% at 298 K and 600 MHz.

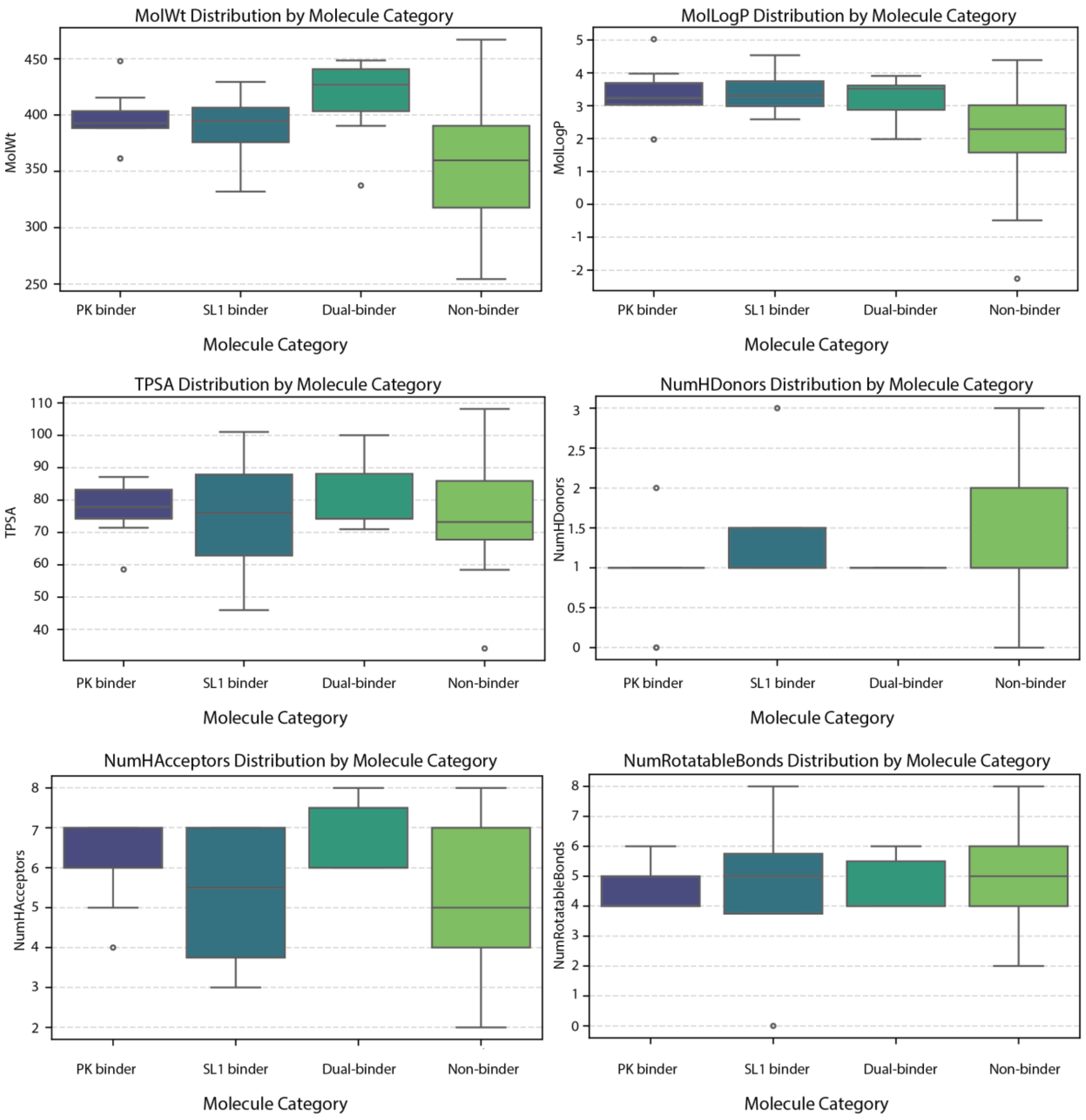

Supplementary Figure 6. **Distribution of physicochemical properties across ligand categories.** The boxplots display key molecular descriptors for each group: PK and SL1 selective binders, dual-binders and non-binders. Shown are molecular weight (MolWt), lipophilicity (MolLogP), topological polar surface area (TPSA), number of hydrogen bond donors (NumHDonors) and acceptors (NumHAcceptors) and rotatable bonds (NumRotatableBonds). Higher median values and for MolWt and MolLogP are observed in the binding categories compared to non-binders, suggesting their potential relevance to RNA interaction. In contrast, TPSA, hydrogen bonding and flexibility parameters show no visual separation.

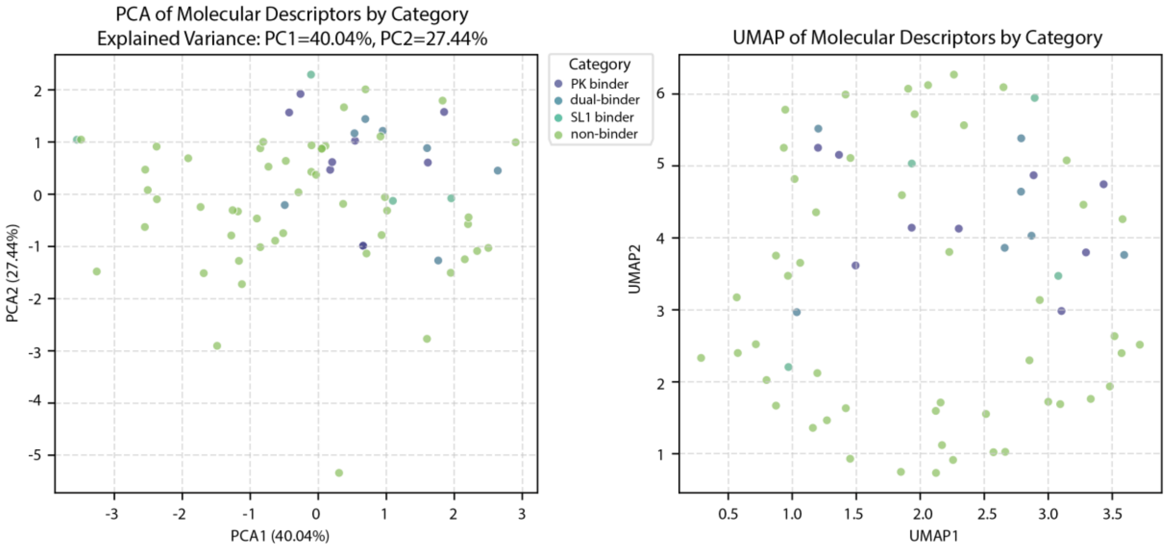

Supplementary Figure 7. **Dimensionality reduction of molecular descriptors by ligand category.** PCA and UMAP projection based on six physicochemical descriptors (MolWt, MolLogP, TPSA, NumHDonors, NumHAcceptors, NumRotatableBonds) calculated for all molecules. Each point represents a compound, color coded by binding category: PK binder in dark blue, dual-binder in blue, SL1 binder in teal and non-binder in green. The first components explain 67.48 % of the total variance. Despite this, no clear separation between categories is observed, in either PCA or UMAP, indicating a high degree of overlap in descriptor space.

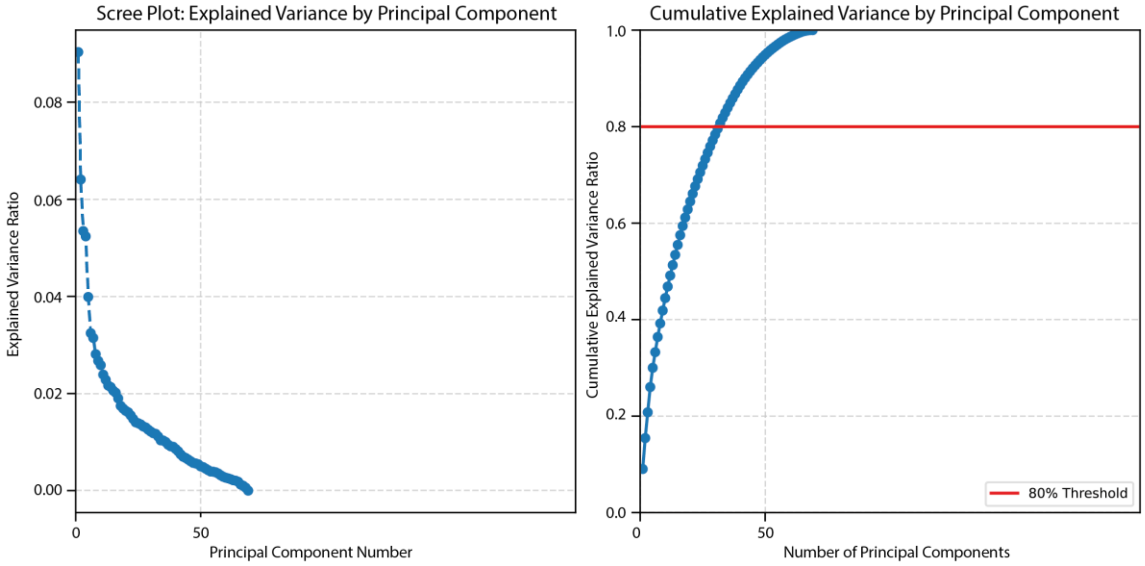

Supplementary Figure 8. **Explained variance of principal components derived from Morgan fingerprint bits.** Scree plot illustrating the proportion of variance explained by each individual principal component. The cumulative variance curve shows the proportion of total variance captured as additional components are included. The red line marks the 80% threshold, requiring 32 components to be reached, which highlights the dataset's high dimensionality.

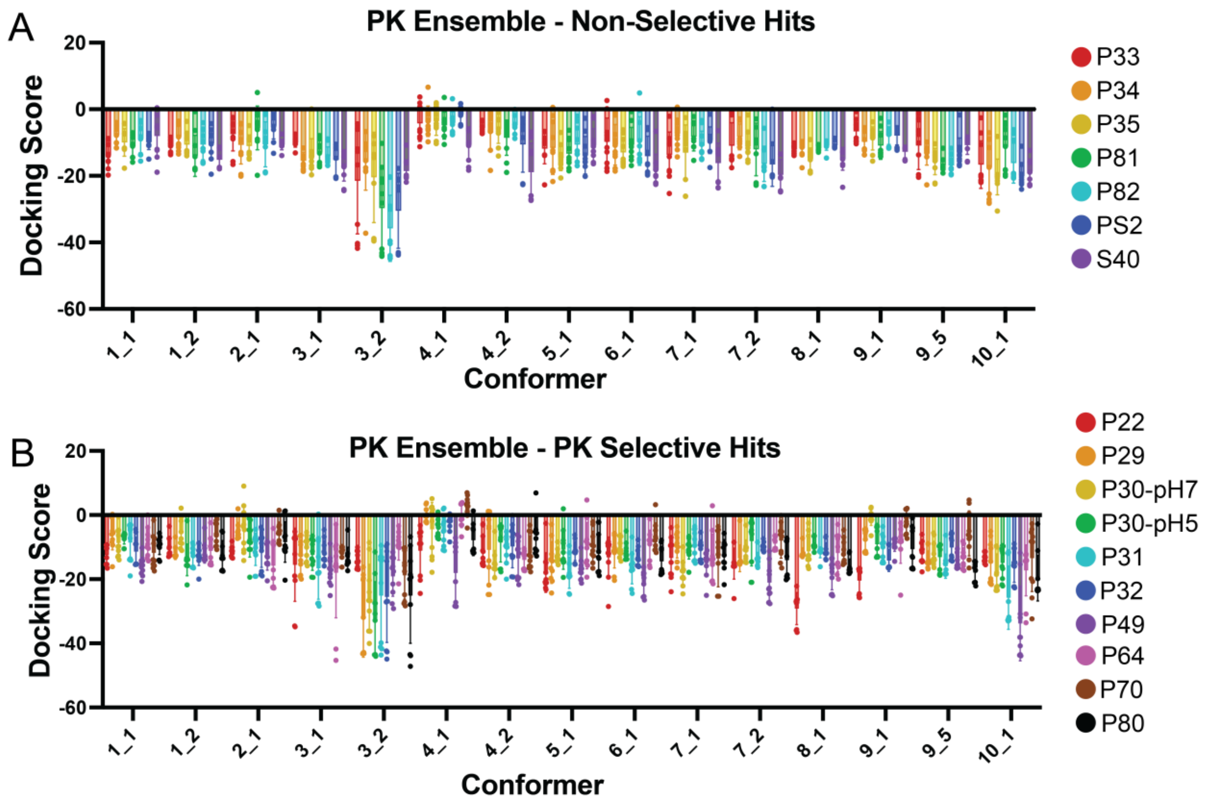

Supplementary Figure 9. Conformer Score analysis for PK ensemble. Scores by conformer, including separate scores if there are multiple pockets per conformer, for all PK non-selective hits (A) and selective hits (B).

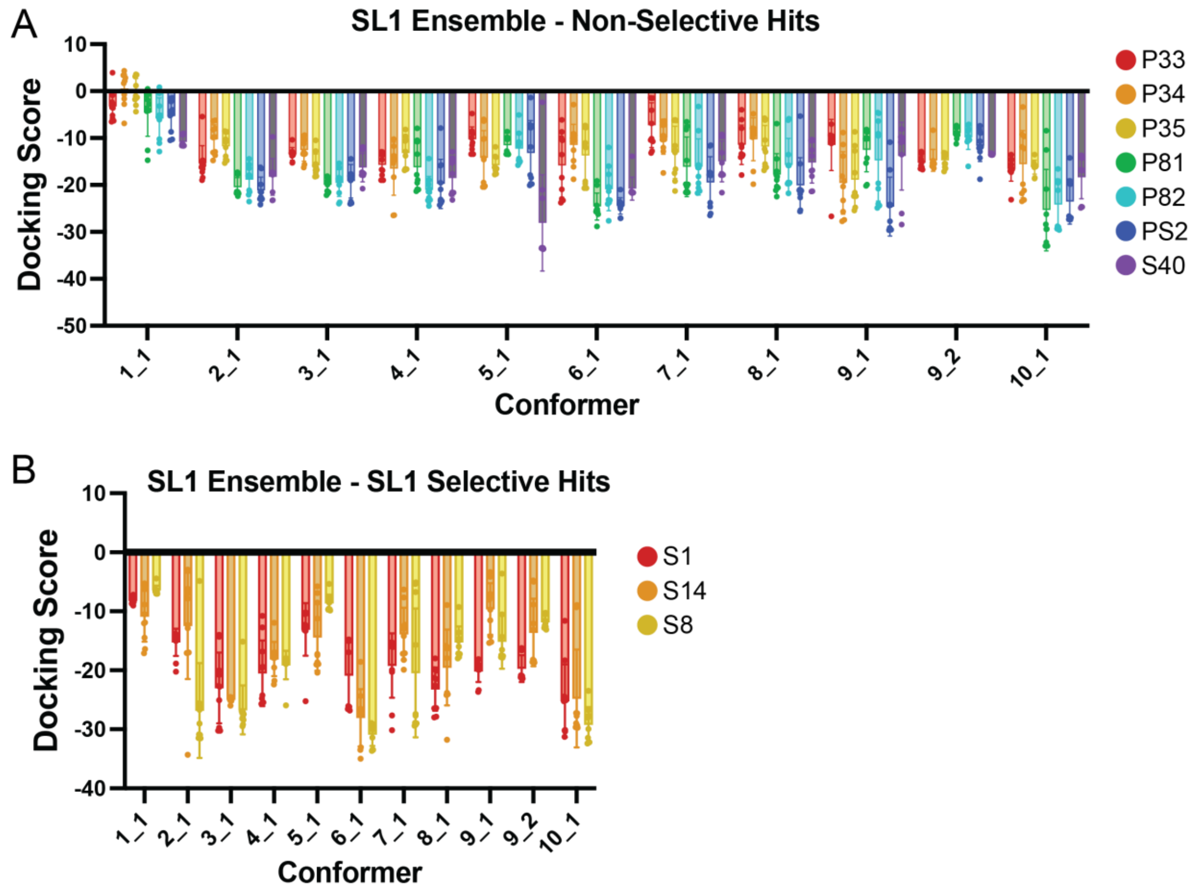

Supplementary Figure 10. Conformer Score analysis for SL1 ensemble. Scores by conformer, including separate scores if there are multiple pockets per conformer, for all SL1 non-selective hits (A) and selective hits (B).

Supporting Tables

Supplementary Table 1. **Compounds ordered after classification as hit in virtual screenings (VS).** The mentioned libraries include information regarding i.) the via VS determined target, ii.) the individual Tier of selection (T1 = Tier1, T2 = Tier2, T3 = Tier3), and iii.) the original library (RNA-focused (RF) or 50K library). The hits were ordered from Life Chemicals Inc. and can be found by the listed ID.

Compound	ID	SMILES	VS hit	NMR hit	SubLib 1	SubLib 2	QC passed [x = yes]?
PS1	F2269-0211	COc1cccc(C(=O)Nc2ccc3c(c2)C(=O)Nc2ccccc2O3)c1OC	PK + SL1		SL1-T2-RF		x
PS2	F6363-0887	COc1ccc(Cl)cc1C(=O)N1CCN(c2cc(Nc3ccccn3)nc(C)n2)CC1	PK + SL1	PK + SL1	SL1-T2-50K	PK-T1-50K	x
P1	F0550-0204	O=C(c1cnc2n(c1=O)CCS2)N1CCN(C(c2ccccc2)c2ccc(Cl)cc2)CC1	PK		SL1-T1-50K		x
P2	F0806-0303	O=C(Nc1cccc(-c2nc3ccccc3[nH]2)c1)c1ccc(C(=O)Nc2cccc(-c3nc4ccccc4[nH]3)c2)s1	PK		SL1-T2-50K		
P3	F0882-1082	CC(C)Oc1ccc(C(=O)Nc2ccccc2-c2nc3ccccc3s2)cc1	PK		SL1-T2-RF		
P4	F1166-0092	CC(C)Oc1ccc(C(=O)Nc2ccccc2-c2nc3ccccc3[nH]2)cc1	PK		PK-T3-50K		
P5	F1957-0484	Cc1nc2nc(O)c(Br)cc2c(C)c1Br	PK	SL1	PK-T1-50K		x
P6	F2158-0821	Cc1ccc(C)c2sc(NCC3CCCO3)nc12	PK		PK-T1-50K		x
P7	F2265-0115	COc1cccc2cc(C(=O)Nc3ccc(-c4nc5ccccc5[nH]4)cc3)oc12	PK		PK-T1-50K		
P8	F2269-0218	COc1cccc(OC)c1C(=O)Nc1ccc2c(c1)C(=O)Nc1ccccc1O2	PK		SL1-T2-RF	PK-T2-RF	
P9	F2269-0314	COc1cccc(C(=O)Nc2ccc3c(c2)C(=O)Nc2cc(C)ccc2O3)c1OC	PK		PK-T2-50K		
P10	F2359-0550	Cc1cc(Cl)ccc1Nc1nc(NCC2CCCO2)nc2nccnc12	PK		SL1-T2-50K		
P11	F2501-0631	COc1cccc(NC(=O)c2cnc(SC)n(C)c2=O)c1	PK		PK-T2-50K		
P12	F2621-0387	C=CCn1ccc2c(OCC(N)=O)cccc2c1=O	PK		SL1-T2-50K	SL1-T1-50K	x
P13	F3309-0408	COc1ccccc1C(=O)N1CCN(c2oc(/C=C/c3ccccc3Cl)nc2C#N)CC1	PK		PK-T3-50K		
P14	F3309-0436	CCOc1ccc(/C=C/c2nc(C#N)c(N3CCN(C(=O)c4ccccc4C)CC3)o2)cc1	PK		PK-T3-50K		
P15	F3376-1659	Cn1c(=O)c2c(nc3n2CCN3CC(N)=O)n(C)c1=O	PK		PK-T3-50K		x
P16	F3379-0434	CCc1nccn1-c1ncnc2c1cnn2Cc1ccccc1Cl	PK		PK-T2-RF		x
P17	F3411-3417	COc1ccccc1-c1cc2c(=O)n(Cc3ccccc3Cl)ccn2n1	PK		SL1-T2-50K		
P18	F5095-0043	COc1cccc(C(=O)N2CCN(c3nccn3C)CC2)c1OC.Cl	PK		PK-T3-50K		x
P19	F5098-0891	O=C(Nc1ccc2c(c1)N(C(=O)C1CC1)CC2)c1ccc(Cl)cc1Cl	PK		PK-T2-50K		
P20	F5530-0950	Cc1nc2ccc(NC(=O)c3cccnc3)cc2c(=O)n1-c1ccc(Cl)cc1	PK		PK-T1-50K		
P21	F5557-0024	O=C(CSc1ncccc1-c1nc2ccccc2[nH]1)NCC1CCCO1	PK		PK-T2-RF		x
P22	F5782-0139	COc1cccc2cc(C(=O)NCC3CCC(c4nc5ccccc5[nH]4)CC3)oc12	PK	PK	PK-T1-50K		x
P23	F5857-6056	COc1ccc(Cl)cc1NC(=O)NCC(C)(O)C1CC1	PK		PK-T2-50K		x
P24	F5857-6814	COc1ccc(Cl)cc1NC(=O)NCC1(O)C=CCCC1	PK		PK-T3-50K		x
P25	F5865-0512	Cc1cc(N2CCN(C(=O)c3nccc(C)n3)CC2)nc(NC2CCCCC2)n1	PK		PK-T3-50K		x
P26	F6221-0175	Cc1nccn1-c1ncc(NC(=O)c2cccc(Oc3ncccn3)c2)cn1	PK		PK-T2-50K	PK-T1-50K	
P27	F6245-5069	COCCn1nc(C2CCCN(C(=O)c3csc(-c4ccccc4)n3)C2)n(C2CC2)c1=O	PK		PK-T1-50K		x
P28	F6252-4128	O=c1c(-c2nc(-c3ccccc3Cl)no2)cccn1Cc1cc(=O)n2ccccc2n1	PK		PK-T2-50K		

P29	F6363-0812	COc1ccccc1C(=O)N1CCN(c2cc(Nc3ccccn3)ncn2)CC1	PK	SL1	PK-T3-50K		x
P30	F6363-0814	O=C(c1ccccc1Cl)N1CCN(c2cc(Nc3ccccn3)ncn2)CC1	PK	PK	PK-T3-50K	PK-T2-50K	x
P31	F6363-0830	O=C(c1ccccn1)N1CCN(c2cc(Nc3ccccn3)ncn2)CC1	PK	PK	PK-T2-50K	PK-T1-50K	x
P32	F6363-0831	O=C(c1ccncc1)N1CCN(c2cc(Nc3ccccn3)ncn2)CC1	PK	PK	PK-T3-50K		x
P33	F6363-0894	COc1ccc(C(=O)N2CCN(c3cc(Nc4cc(C)ccn4)nc(C)n3)CC2)cc1OC	PK	PK + SL1	PK-T1-50K		x
P34	F6363-0897	Cc1ccnc(Nc2cc(N3CCN(C(=O)CCc4ccccc4)CC3)nc(C)n2)c1	PK	PK + SL1	PK-T2-50K		x
P35	F6363-0906	Cc1ccnc(Nc2cc(N3CCN(C(=O)c4cnccn4)CC3)nc(C)n2)c1	PK	PK + SL1	PK-T3-50K		x
P36	F6412-1251	COc1ccccc1OCC#CCNC(=O)c1ccc(Cn2ccnc2)cc1	PK		PK-T2-50K		x
P37	F6414-0059	Cn1cccc(C(=O)NCC(O)c2ccc3c(c2)CCO3)c1=O	PK		PK-T1-50K		x
P38	F6414-0065	O=C(NCC(O)c1ccc2c(c1)CCO2)c1ccccc1Cl	PK		PK-T2-50K	PK-T1-50K	x
P39	F6414-0098	O=C(NCC(O)c1ccc2c(c1)CCO2)c1cccc2ccccc12	PK		PK-T1-50K		x
P40	F6414-0192	COc1ccc(Br)c(C(=O)NCC(O)c2ccc3c(c2)CCO3)c1	PK		PK-T1-50K		x
P41	F6418-2631	COc1ccc(CC(=O)NCc2ccc(C(O)c3ccccc3)s2)cc1OC	PK		PK-T1-50K		x
P42	F6436-1453	O=C(Nc1ccc2c(c1)N(C(=O)C1CC1)CC2)c1cncc(Br)c1	PK		PK-T3-50K		
P43	F6438-3256	COc1nc(CNC(=O)c2cccn(C)c2=O)nc(N2CCCC2)n1	PK		PK-T2-50K		x
P44	F6438-3275	COc1nc(CNC(=O)c2ccc(Cn3ccnc3)cc2)nc(N2CCCC2)n1	PK		PK-T2-50K		x
P45	F6438-3553	CN(C)c1nc(CNC(=O)c2ccc(Cn3ccnc3)cc2)nc(N(C)C)n1	PK		PK-T1-50K		x
P46	F6446-0206	CC(=O)Nc1ccc(CC(=O)NC2CCN(c3ccccn3)C2)cc1	PK		PK-T2-50K		x
P47	F6448-2065	Cn1cccc(C(=O)Nc2cnn(CC3COc4ccccc4O3)c2)c1=O	PK		PK-T3-50K		x
P48	F6448-2066	COc1cccc2cc(C(=O)Nc3cnn(CC4COc5ccccc5O4)c3)oc12	PK		PK-T3-50K		
P49	F6448-2068	O=C(Nc1cnn(CC2COc3ccccc3O2)c1)c1ccc(Cn2ccnc2)cc1	PK	PK	PK-T3-50K		x
P50	F6448-2249	Cc1ccc(N2CC(C(=O)Nc3cnn(CC4COc5ccccc5O4)c3)CC2=O)cc1C	PK		PK-T1-50K		
P51	F6451-1625	COC(CNC(=O)c1cccn(C)c1=O)c1ccccc1C	PK		PK-T2-50K		x
P52	F6469-1732	COc1cccc(N2CC(C(=O)Nc3ccccc3-c3cn4ccccc4n3)CC2=O)c1	PK		PK-T3-50K		
P53	F6469-2004	Cc1cccn2cc(-c3ccccc3NC(=O)Cc3noc4ccccc34)nc12	PK		PK-T1-50K		
P54	F6473-3854	CC(C)(C)c1ccc(NC(=O)N2CC(N3C(=O)CCC3=O)C2)cc1	PK		PK-T3-50K		
P55	F6482-6039	O=C(NCc1ccnc(-c2ccncc2)c1)c1ccc(Cn2ccnc2)cc1	PK		PK-T3-50K		x
P56	F6495-5852	Cc1cc(C)n(-c2cc(N3CC(C(=O)NCCn4ccnc4C)C3)ncn2)n1	PK		PK-T2-50K		x
P57	F6515-4015	O=C(NCc1cncc(-c2cccnc2)c1)c1ccc(Cn2ccnc2)cc1	PK		PK-T2-50K		x
P58	F6570-2537	COc1cccc(N2CC(C(=O)Nc3ccccc3-c3cn4c(n3)CCCC4)CC2=O)c1	PK		PK-T3-50K		
P59	F6570-2718	O=C(Nc1ccccc1-c1cn2c(n1)CCCC2)c1cc(=O)n(-c2ccccc2)c(=O)[nH]1	PK		PK-T3-50K		
P60	F6573-0170	Cn1nccc1-c1ccc(CNC(=O)c2ccc3[nH]cnc3c2)cn1	PK		PK-T2-50K		x
P61	F9995-2687	CCc1ccc(NC(=O)N2CC=C(c3c[nH]c4ccccc34)CC2)cc1	PK		PK-T3-50K		
P62	F0922-0787	O=C(CCn1c(=O)c2ccccc2n(Cc2ccccc2F)c1=O)N1CCc2ccccc2C1	PK		SL1-T2-50K		
P63	F1883-0783	O=C(Nc1ccc(F)c(F)c1)c1oc2ccccc2c1NC(=O)C1CCCO1	PK		PK-T3-50K		
P64	F2255-0696	NC(=O)C1CCN(c2ccc3cccc(OCc4ccc(C(F)(F)F)cc4)c3n2)CC1	PK	SL1	SL1-T2-50K		x
P65	F2442-0300	Cc1nc(Oc2ccccc2)cc(N2CCN(C(=O)c3cccc(C(F)(F)F)c3)CC2)n1	PK		SL1-T3-50K		
P66	F2691-0113	Nc1c(C(=O)Nc2cccc(C(F)(F)F)c2)nnn1Cc1ccc(Cl)cc1	PK		PK-T1-50K		

P67	F3309-0405	N#Cc1nc(/C=C/c2ccccc2Cl)oc1N1CCN(C(=O)c2ccccc2F)CC1	PK		PK-T3-50K		
P68	F3350-0945	Cc1cccc(NC(=O)c2cnc3c(C(F)(F)F)cccc3c2O)n1	PK		PK-T3-50K		
P69	F3367-0114	Cc1cccc(NC(=O)c2cnc3ccc(F)cc3c2O)n1	PK		PK-T2-50K		
P70	F5127-0821	CN(C)CCN(C(=O)c1ncccn1)c1nc2ccc(OC(F)(F)F)cc2s1.Cl	PK	PK	PK-T2-50K		x
P71	F5564-0059	Cc1nc(Nc2ccc(NC(=O)c3ccc(F)cc3Cl)cc2)cc(-n2cccn2)n1	PK		PK-T1-50K		
P72	F5596-0842	O=C(Cn1cnc2ccccc21)N1CCC(c2nnc(-c3ccc(F)cc3)o2)CC1	PK		PK-T1-50K		
P73	F5788-2252	NC(=O)CN1CCN(c2ccc(F)c(F)c2)C1=O	PK		PK-T2-50K	PK-T1-50K	x
P74	F6221-0037	O=C(Nc1cnc(-n2ccnc2)nc1)c1c(F)cccc1Cl	PK		PK-T2-50K	PK-T1-50K	x
P75	F6221-0039	O=C(Nc1cnc(-n2ccnc2)nc1)c1cccc(C(F)(F)F)c1	PK		PK-T2-50K		
P76	F6252-4142	O=c1c(-c2nc(-c3ccccc3Cl)no2)cccn1Cc1noc(-c2cccc(F)c2)n1	PK		PK-T3-50K		
P77	F6252-5108	O=C(c1cn(CC(O)c2ccccc2F)nn1)N1CCN(C2CCCC2)CC1	PK		PK-T3-50K		x
P78	F6363-0815	O=C(c1c(F)cccc1F)N1CCN(c2cc(Nc3ccccn3)ncn2)CC1	PK		PK-T3-50K		
P79	F6363-0816	O=C(c1cccc(C(F)(F)F)c1)N1CCN(c2cc(Nc3ccccn3)ncn2)CC1	PK		PK-T3-50K		x
P80	F6363-0882	Cc1nc(Nc2ccccn2)cc(N2CCN(C(=O)c3cccc(F)c3)CC2)n1	PK	PK	PK-T1-50K		x
P81	F6363-0883	Cc1nc(Nc2ccccn2)cc(N2CCN(C(=O)c3c(F)cccc3Cl)CC2)n1	PK	PK + SL1	PK-T2-50K		x
P82	F6363-0884	Cc1nc(Nc2ccccn2)cc(N2CCN(C(=O)c3ccccc3C(F)(F)F)CC2)n1	PK	PK + SL1	PK-T1-50K		x
P83	F6411-4523	O=C(CCn1nc(-c2ccc(F)cc2)ccc1=O)N1CCN(c2ccccn2)CC1	PK		PK-T1-50K		
P84	F6414-3173	COc1ccc(C(O)CNC(=O)c2ccccc2C(F)(F)F)cc1OC	PK		PK-T1-50K		
P85	F6436-3709	O=C(CCc1ccccc1F)NCC(O)c1ccc2c(c1)CCO2	PK		PK-T2-50K		
P86	F6469-2183	Cc1cccn2cc(-c3ccccc3NC(=O)c3ccc(C(F)(F)F)nc3)nc12	PK		PK-T3-50K		
P87	F6495-4402	O=C(C1CN(c2cc(-n3ccnc3)ncn2)C1)N1CCN(c2ccc(F)c(Cl)c2)CC1	PK		PK-T2-50K		
P88	F6523-3982	O=C(NCC1CCCO1)N1CCc2nc3ccc(F)cn3c(=O)c2C1	PK		PK-T2-50K		x
S1	F0657-0459	COc1cccc(C(=O)Nc2cccc(-c3nc4ncccn4c3C)c2)c1OC	SL1	PK	PK-T3-50K		x
S2	F0806-0325	O=C(COCC(=O)Nc1cccc(-c2nc3ccccc3[nH]2)c1)Nc1cccc(-c2nc3ccccc3[nH]2)c1	SL1		PK-T2-RF		
S3	F2199-0382	COc1cccc(-c2noc(CN3C(=O)c4ccccc4C3=O)n2)c1OC	SL1		PK-T3-50K		
S4	F2269-0062	COc1cccc(C(=O)Nc2ccc3c(c2)C(=O)N(C)c2ccccc2O3)c1	SL1		PK-T3-50K		
S5	F2269-0117	CCN1C(=O)c2cc(NC(=O)c3cccc(OC)c3OC)ccc2Oc2ccccc21	SL1		SL1-T2-RF		
S6	F2269-0497	COc1cccc(C(=O)Nc2ccc3c(c2)C(=O)N(C)c2cc(C)ccc2O3)c1OC	SL1		PK-T2-RF		
S7	F2318-0076	COc1ccc(Cl)cc1C(=O)N1CCN(c2ccc(-n3cccn3)nn2)CC1	SL1		SL1-T2-RF		x
S8	F2442-0239	Cc1nc(OC(C)C)cc(N2CCN(C(=O)c3cccc4ccccc34)CC2)n1	SL1	PK	PK-T1-50K		x
S9	F2501-0641	COc1cccc(NC(=O)c2cnc(SCC(=O)NC3CCCC3)n(C)c2=O)c1	SL1		PK-T3-50K		
S10	F3382-8198	COc1cccc(-c2nc(Cn3c(=O)n(CC4CCCO4)c(=O)c4ccccc43)c(C)o2)c1OC	SL1		PK-T1-50K		
S11	F3406-5426	O=C1CCCN1c1ccc(Cn2nc3ccc(-c4ccccc4)nn3c2=O)cc1	SL1		SL1-T2-50K		
S12	F3406-5477	COc1cccc(-c2noc(Cn3nc4ccc(-c5ccc(C)c(C)c5)nn4c3=O)n2)c1OC	SL1		SL1-T3-50K		
S13	F5238-0027	O=C(NCCNc1ccc(Nc2ccncc2)nn1)c1ccccc1Cl	SL1		PK-T2-50K		
S14	F5238-0055	COc1ccc(Cl)cc1C(=O)NCCNc1ccc(Nc2ccncc2)nn1	SL1	SL1	SL1-T1-50K		x
S15	F5283-0096	COc1ccc(Cl)cc1C(=O)NCCn1ncccc1=O	SL1		SL1-T2-50K		x
S16	F5971-0062	COCC(C)(O)CNC(=O)c1cccn(C)c1=O	SL1		SL1-T1-50K		x

S17	F6064-0701	COc1ccc(Cl)cc1C(=O)N1CC(c2nc(-c3ncccn3)no2)C1	SL1		SL1-T2-50K		x
S18	F6089-9681	COCC(=O)N1CCCc2ccc(NC(=O)NCC3CCCO3)cc21	SL1		SL1-T2-50K		x
S19	F6125-2352	CC(=O)N1CC(C(=O)Nc2ccc3sc(C)nc3c2)C1	SL1		SL1-T2-50K		x
S20	F6235-0058	O=C(c1cccc2ccccc12)N1CCN(c2ccc(-n3ccnc3)nn2)CC1	SL1		SL1-T2-50K		x
S21	F6235-0188	O=C(Cn1cnc2ccccc21)N1CCN(c2ccc(-n3ccnc3)nn2)CC1	SL1		SL1-T1-50K		x
S22	F6252-3887	Cc1noc(-c2cccn(Cc3cc(=O)n4ccccc4n3)c2=O)n1	SL1		SL1-T2-50K		x
S23	F6262-1842	CCOc1cc(NC(=O)C2CC(=O)N(c3cccc(OC)c3)C2)ncn1	SL1		SL1-T2-50K		x
S24	F6438-3544	CN(C)c1nc(CNC(=O)COCc2ccccc2)nc(N(C)C)n1	SL1		SL1-T1-50K		
S25	F6442-5822	COC1CCN(C2CCN(C(=O)COc3cccc(C)c3)CC2)C1	SL1		SL1-T1-50K		x
S26	F6469-2165	COc1ccccc1-c1cc(C(=O)Nc2ccccc2-c2cn3cccc(C)c3n2)[nH]n1	SL1		SL1-T1-50K		
S27	F6475-0919	O=C(NCc1ccc(N2CCOCC2)nc1)c1cccnc1OC1CCOC1	SL1		SL1-T1-50K		x
S28	F6475-1234	O=C(Nc1ccc2c(c1)OCCO2)c1ccnc(OC2CCOC2)c1	SL1		SL1-T2-50K		x
S29	F6493-1243	Cc1nc(-c2cccc(NC(=O)COc3ccnc4c3CCCC4)c2)cs1	SL1		SL1-T3-50K		x
S30	F6493-1268	Cc1nc(NC(=O)COc2ccnc3c2CCCC3)sc1C	SL1		SL1-T2-50K		x
S31	F6495-4584	Cc1ccc(=O)n(CCNC(=O)C2CN(c3cc(-n4ccnc4)ncn3)C2)n1	SL1		SL1-T1-50K		x
S32	F6495-5356	O=C(Nc1ccc2c(c1)OCCO2)C1CN(c2cc(-n3cccn3)ncn2)C1	SL1		SL1-T1-50K		
S33	F6514-3781	Cn1cccc(C(=O)N2CC(n3cc(COc4ccccc4)nn3)C2)c1=O	SL1		SL1-T2-50K		x
S34	F6514-3830	COc1ccc(Cl)cc1C(=O)N1CC(n2cc(COc3ccccc3)nn2)C1	SL1		SL1-T3-50K		x
S35	F6514-4058	COc1cc(=O)n(C)cc1C(=O)N1CC(n2cc(COc3ccccc3)nn2)C1	SL1		SL1-T2-50K		x
S36	F6514-4372	COc1cccc2cc(C(=O)N3CC(n4cc(CN5CCCC5=O)nn4)C3)oc12	SL1		SL1-T2-50K		x
S37	F6514-4384	CC1(C)Cc2cccc(OCC(=O)N3CC(n4cc(CN5CCCC5=O)nn4)C3)c2O1	SL1		SL1-T2-50K		x
S38	F6550-6090	CC1(C)Cc2cccc(OCC(=O)N3CC(Nc4cccnn4)C3)c2O1	SL1		SL1-T1-50K		x
S39	F6550-6757	O=C(c1ccc(Br)o1)N1CC(Nc2ccncn2)C1	SL1		SL1-T1-50K		x
S40	F6550-6822	O=C(c1csc(-c2ccccc2)n1)N1CC(Nc2ccncn2)C1	SL1	PK + SL1	SL1-T2-50K		x
S41	F6562-2382	COc1ccc(Cl)cc1C(=O)NCc1ccnc(N2CCCC2=O)c1	SL1		SL1-T1-50K		x
S42	F0919-6466	O=c1c2ccccc2n(Cc2cc(=O)n3cc(Cl)ccc3n2)c(=O)n1-c1ccc(F)c(Cl)c1	SL1		PK-T3-50K		
S43	F6514-3810	O=C(c1ccc(F)cc1Cl)N1CC(n2cc(COc3ccccc3)nn2)C1	SL1		SL1-T2-50K		
S44	F6514-4301	O=C1CCCN1Cc1cn(C2CN(C(=O)c3ccccc3F)C2)nn1	SL1		SL1-T2-50K		

Supplementary Table 2. **Chemical shift perturbations (CSPs) in ppm used for the mapping shown in Figure 7.** Listed are SL1's pyrimidine resonances detected by 2D-^{1}H,^{1}H TOCSYs and PK's imino resonances detected in the ^{1}H,^{15}N-TROSYs experiments. Unassignable resonances (due to line-broadening or signal overlap) are marked with -.

		^{1}H,^{1}H CSPs [ppm]	
	**Assignment**	**S14**	**P35**
**SL1**	**U9-H5H6**	0.009	0.035
	**U10-H5H6**	0.014	0.026
	**U11-H5H6**	0.044	0.079
	**U13-H5H6**	0.050	-
	**C15-H5H6**	0.007	0.017
	**C16-H5H6**	0.008	0.025
	**U17-H5H6**	0.007	0.008
	**U18-H5H6**	0.004	0.010
	**C19-H5H6**	0.005	0.031
	**C20-H5H6**	0.007	0.029
	**C21-H5H6**	0.004	0.040
	**U25-H5H6**	0.007	0.038
	**C28-H5H6**	0.079	0.277
	**C32-H5H6**	-	-
	**C33-H5H6**	0.006	0.020
	**C34-H5H6**	0.013	0.022

		^{1}H,^{15}N CSPs [ppm]	
	**Assignment**	**P80**	**P35**
**PK**	**G475-H1N1**	-	-
	**G478-H1N1**	0.002	0.014
	**U479-H3N3**	0.002	0.007
	**G480-H1N1**	0.002	0.012
	**U481-H3N3**	0.013	0.003
	**G484-H1N1**	0.009	0.029
	**G489-H1N1**	0.009	-
	**U496-H3N3**	0.002	0.014
	**U497-H3N3**	0.005	0.010
	**G503-H1N1**	0.002	-
	**G505-H1N1**	-	-
	**G508-H1N1**	0.012	-
	**G513-H1N1**	-	-
	**G525-H1N1**	0.008	-
	**U529-H3N3**	0.002	0.028
	**G531-H1N1**	0.018	-
	**G539-H1N1**	0.002	0.003
	**G540-H1N1**	0.005	0.018

Supplementary Table 3. **Mean physicochemical descriptor values and one-way ANOVA results across ligand categories.** Summarized average values for six key molecular descriptors: molecular weight (MolWt), lipophilicity (MolLogP), topological polar surface area (TPSA), number of hydrogen bond donors (NumHDonors) and acceptors (NumHAcceptors) and rotatable bonds (NumRotatableBonds) across the four ligand categories. F-statistics and p-values from a one-way ANOVA are included to assess whether differences between group means were statistically significant. For MolWt (p=0.04) and MolLogP (p=0.005), the differences are statistically significant (p < 0.05). For the remaining descriptors, no significant differences were detected.

**Descriptor**	**Mean PK binder**	**Mean Dual-binder**	**Mean SL1 binder**	**Mean Non-binder**	**F-Statistic**	**P-Value**
MolWt [g/mol]	395.093	414.452	387.685	354.026	4.978	0.004
MolLogP	3.302	3.197	3.429	2.174	4.627	0.005
TPSA [Å²]	77.099	81.426	74.75	75.614	0.393	0.759
NumHDonors	0.889	1	1.5	1.122	0.628	0.599
NumHAcceptors	6.111	6.714	5.25	5,367	2.016	0.12
NumRotatableBonds	4.667	4.714	4.5	4,796	0.064	0.978

Supplementary Table 4. **Fingerprint bits with high frequency in RNA-binding ligands.** Listed are Morgen fingerprint bits (ECFP4, radius = 2, 2048 bits) that occur frequently in ligands binding to PK and/or SL1 RNA structures, while being largely absent in non-binding compounds. For each bit, the relative frequency within each ligand category is shown, along with representative compound IDs associated with each bit. Bits were selected based on a frequency threshold of > 50 % in at least one binder group and < 10 % in non-binders.

Fingerprint Bit Index	PK Binders Frequency	SL1 Binders Frequency	Non-binder Frequency	Associated binder
430	0.63	0.73	0	P29, P30, P31, P32, P33, P34, P35, P80, P81, P82, S14, PS2
491	0.63	0.73	0	P29, P30, P31, P32, P33, P34, P35, P80, P81, P82, S14, PS2
571	0.5	0.64	0	P32, P33, P34, P35, P80, P81, P82, S8, S14, PS2
477	0.56	0.64	0.04	P29, P30, P31, P32, P33, P34, P35, P64, P80, P81,P82,
719	0.56	0.55	0.02	P29, P30, P31, P32,P33, P34, P35, P80, P81, P82,
1275	0.5	0.55	0.02	P33, P34, P35, P80, P81, P82, S8, PS2
2018	0.5	0.45	0.02	P29, P30, P31, P32, P33, P34, P35, P70, PS2
